# Compartment-specific multiomic profiling identifies SRC and GNAS as candidate drivers of epithelial-to-mesenchymal transition in ovarian carcinosarcoma

**DOI:** 10.1038/s41416-023-02508-3

**Published:** 2023-12-14

**Authors:** C. Simon Herrington, Ailsa J. Oswald, Lorna J. Stillie, Ian Croy, Michael Churchman, Robert L. Hollis

**Affiliations:** 1 The Nicola Murray Centre for Ovarian Cancer Research, Cancer Research UK Scotland Centre, Institute of Genetics and Cancer, University of Edinburgh, Edinburgh, UK; 2 Cancer Research UK Scotland Centre and Cancer Research UK Beatson Institute, Institute of Cancer Sciences, University of Glasgow, Glasgow, UK

**Keywords:** Ovarian cancer, Transcriptomics

## Abstract

**Background:**

Ovarian carcinosarcoma (OCS) is an exceptionally aggressive and understudied ovarian cancer type harbouring distinct carcinomatous and sarcomatous compartments. Here, we seek to identify shared and compartment-specific events that may represent potential therapeutic targets and candidate drivers of sarcomatous compartment formation through epithelial-to-mesenchymal transition (EMT).

**Methods:**

We performed multiomic profiling (exome sequencing, RNA-sequencing, microRNA profiling) of paired carcinomatous and sarcomatous components in 12 OCS cases.

**Results:**

While paired sarcomatous and carcinomatous compartments demonstrate substantial genomic similarities, multiple loci are recurrently copy number-altered between components; regions containing *GNAS* and *SRC* are recurrently gained within the sarcomatous compartment. *CCNE1* gain is a common event in OCS, occurring more frequently than in high grade serous ovarian carcinoma (HGSOC). Transcriptomic analysis suggests increased MAPK activity and subtype switching toward poor prognosis HGSOC-derived transcriptomic subtypes within the sarcomatous component. The two compartments show global differences in microRNA profiles, with differentially expressed microRNAs targeting EMT-related genes (*SIRT1*, *ZEB2*) and regulators of pro-tumourigenic pathways (TGFβ, NOTCH); chrX is a highly enriched target of these microRNAs and is also frequently deleted across samples. The sarcomatous component harbours significantly fewer CD8-positive cells, suggesting poorer immune engagement.

**Conclusion:**

*CCNE1* gain and chrX loss are frequent in OCS. *SRC* gain, increased *GNAS* expression and microRNA dysregulation represent potential mechanisms driving sarcomatous compartment formation.

## Background

Ovarian carcinosarcoma (OCS) is an exceptionally aggressive ovarian cancer type, with a median survival of 12–24 months [1, 2]. OCS is biphasic, harbouring malignant epithelial (carcinomatous) and malignant mesenchymal (sarcomatous) compartments [2, 3]. Historically, this led to their classification as ovarian sarcomas. However, we now recognise that OCS is of epithelial origin, accounting for 2–4% of ovarian carcinomas, with the sarcomatous population having undergone complete epithelial-to-mesenchymal transition (EMT) from the carcinomatous population [2, 4].

Most OCS cases have a carcinomatous component that is of high grade serous (HGS) type, though a significant proportion (~20%) are of endometrioid type [1, 3]. However, in recent years it has become apparent that OCS is not simply a disease variant of high grade serous ovarian carcinoma (HGSOC). Compared with HGSOC, OCS is associated with significantly poorer survival [1, 5, 6], is significantly more intrinsically chemoresistant (response rate 25–65%) [1, 6, 7], is more frequently diagnosed at an earlier stage (15% FIGO I, 10% FIGO II) [1, 5], and affects women at a significantly older age (median 66–70 years) [1, 5, 6].

The distinct histopathological appearance and unique clinical characteristics of OCS highlight the need for disease-specific investigations of OCS to improve our understanding of its behaviour and underlying biology. However, despite its aggressive behaviour, OCS has received little research attention to date [2, 4]. Our understanding of molecular similarities and differences between the two compartments is also poor. This is of acute relevance in the context of molecular therapeutics; therapies targeting molecular events shared between both compartments may be expected to demonstrate greater activity than those targeting molecules that are only perturbed in one of the two malignant cell populations. Moreover, little is known about which molecular events may drive the carcinomatous component to undergo EMT and form the sarcomatous population.

A small number of OCS cases have undergone genomic characterisation, most commonly by targeted sequencing [8]. These data have revealed frequent *TP53* mutation but few recurrent mutational events [9]. Little is known about the copy number landscape of OCS, other than that extensive copy number disruption appears common [8]. There is a particular scarcity of transcriptomic characterisation in OCS, and there has been little investigation of whether either malignant compartment is actively engaged by the immune system [2, 4]. Moreover, the microRNA expression landscape in OCS remains completely unexplored.

More comprehensive studies have been performed in uterine carcinosarcoma, which has sometimes included a small number of OCS samples in combined studies of gynaecological carcinosarcoma, leading to the identification of HER2, EFGR and PDGFR as potential therapeutic targets [2, 10, 11]. However, these have been dominated by the large numbers of uterine carcinosarcoma samples, shedding little light on the specific biology of OCS. It is well established that cancers of tubo-ovarian origin demonstrate distinct clinical and molecular behaviour to those of similar histologies arising in the uterus [12**–**16], and the limited available data show global differences in the molecular landscape of OCS and uterine carcinosarcoma [9, 17].

Here, we report comprehensive genomic, transcriptomic and microRNA profiling of paired carcinomatous and sarcomatous compartments in a series of OCS cases, alongside an assessment of tumour-infiltrating immune cell burden.

## Materials and Methods

### Case identification and inclusion criteria

The study cohort comprised paired carcinomatous and sarcomatous FFPE tumour samples from 12 OCS cases with carcinomatous components of the high grade serous type identified as part of a recent cross-sectional study of OCS [1]. From 82 pathologically-confirmed OCS cases identified in this previous work, 65 had carcinomatous components of high grade serous type (all confirmed WT1 positive) [1]; from these 65 cases, 12 were selected for multiomic characterisation based on the availability of paired samples containing pure carcinomatous (confirmed cytokeratin positive) and pure sarcomatous (confirmed vimentin positive) malignant cells with sufficient material for molecular analysis (Table S1). 7 cases contained heterologous sarcomatous elements (4 chondrosarcoma confirmed by S100 immunohistochemistry, 3 rhabdomyosarcoma confirmed by immunohistochemistry for myogenin and desmin [1]); definitively heterologous regions were avoided during molecular profiling.

### Ethical approval

Ethical approval was obtained from the Lothian NRS Human Annotated Bioresource (reference 15/ES/0094-SR1330). All participants gave written informed consent or had consent waived by the ethics committee due to the retrospective nature of the study; this study was performed in accordance with the Declaration of Helsinki.

### Immunohistochemistry

4 µm FFPE sections were used for immunohistochemical (IHC) staining on the Leica BOND III Autostainer (Leica Biosystems) using IHC protocol F. CD3 and CD8 IHC was performed using Leica ready-to-use CD3 (clone LN10, pre-diluted, Leica Biosystems #PA0553) and CD8 (clone 4B11, pre-diluted, Leica Biosystems, #PA0183) mouse monoclonal antibodies. Vimentin and pan-cytokeratin IHC was performed with Leica ready-to-use vimentin (clone V9, pre-diluted, Leica Biosystems #PA0640) and multi-cytokeratin (clone AE1/AE3, pre-diluted, Leica Biosystems #PA0909) monoclonal mouse antibodies as previously described [1]. CD3, CD8, vimentin and multi-cytokeratin staining were performed on carcinomatous and sarcomatous samples from all 12 cases.

### Isolation of carcinomatous and sarcomatous samples

H&E-stained slides from all available FFPE tumour blocks were assessed for the presence of regions containing only carcinomatous or sarcomatous malignant populations. Corresponding slides, stained for vimentin and pan-cytokeratin, were used to confirm the purity of the respective tumour areas. H&E slides were then marked to identify the pure sarcomatous and pure carcinomatous regions, which were then used as a guide for the macrodissection of sequential 10 µm FFPE sections.

### Genomic profiling

Genomic profiling was performed by whole exome sequencing of DNA extracted from matched sarcomatous and carcinomatous samples. DNA extraction was performed using the Qiagen QIAamp DNA FFPE tissue kit (Qiagen, #56404) with deparaffinization solution (Qiagen, #19093) according to the manufacturer’s instructions.

Whole exome libraries were prepared using the Illumina TruSeq Exome Library Prep kit (Illumina, FC-150–1002) according to the manufacturer’s protocol, modified for FFPE DNA (Supplement A), and sequenced using the NextSeq2000 (Illumina). Exome sequencing data were processed using the bcbio nextgen workflow (v1.2.4) for tumour-only sequencing: reads were aligned to the human reference genome (GRCh38) using bwa (v0.7.17) and duplicates were marked prior to base quality score recalibration with GATK v4.1.9. The median per-sample on-target coverage was 109X. Variant calling was performed using a majority vote system from three callers (VarDict 2019.06.04, Mutect2 and Freebayes 1.1.0.46). Called variants were annotated using the ensemble variant effect predictor (VEP v102) and filtered to remove common variation and retain likely functional variants at a variant allele frequency of ≥0.1 (Supplement A).

### Genome-wide copy number analysis

Genome-wide copy number data were derived from aligned BAM files using the CopywriteR R package to calculate relative copy number estimates at 20 kb genomic intervals [18] (Supplement A). Median relative log2 copy number ratios of intervals spanning each chromosome arm were calculated; a median of 0.5 and -0.5 were used as thresholds for chromosome arm-level gains and losses, respectively. Copy number estimates across genomic intervals were compared between samples using Spearman’s rank-sum test to produce a matrix of per-sample correlations. This matrix underwent hierarchical clustering using Euclidean distance and Ward’s linkage. Recurrent copy number changes between sarcomatous and sarcomatous regions were identified using paired Mann–Whitney *U*-tests of quantified copy number estimates across the genomic intervals (Supplement A).

### Targeted *CCNE1* copy number assessment

Copy number of *CCNE1*, encoding cyclin E1, was assessed in each compartment across the 12 OCS cases due to the association of *CCNE1* gain with poor outcome and chemoresistance in HGSOC [2, 4]. *CCNE1* copy number was quantified by TaqMan qPCR (ThermoFisher Scientific, Hs07158517_cn) using NA12878 DNA as a calibrator (copy number = 2) within CopyCaller v2.0 (Thermofisher Scientific). Samples with ≥4 copies of *CCNE1* were considered to be *CCNE1* gained. A comparator cohort of 362 HGSOC cases, characterised by the same assay in a previous study [19], was used to assess differences in *CCNE1*-gain frequency in OCS versus HGSOC.

### MicroRNA profiling

MicroRNA profiling was performed using the HTG Molecular EdgeSeq microRNA Whole Transcriptome Assay (HTG Molecular) (Supplement B). Samples were lysed directly into the target capture reaction and libraries were sequenced using a NextSeq (Illumina). The HTG EdgeSeq Parser was used to align FASTQ files to the probe list and produce raw per-target count data. The median total counts per sample was 13.6 M (range 6.0–17.7 M).

Unsupervised analysis of microRNA expression profiles was performed by principal component analysis. Significantly differentially expressed microRNAs between carcinomatous and sarcomatous compartments were identified using a paired design matrix within the EdgeR R package (Supplement B). Hierarchical clustering was used to identify clusters of differentially expressed microRNAs, which were then annotated for significantly enriched target genes using miEAA version 2.0 [20] (Supplement B). Identified gene targets were mapped to significantly enriched pathways against the Molecular Signatures 2020 Database [21] using the enrichR R package [22].

### Transcriptomic profiling

mRNA profiling was performed by Lexogen Quantseq 3’ mRNA-Seq (Lexogen Inc, #015) (Supplement C). Generated libraries were sequenced using the NextSeq 550 (Illumina), achieving a mean coverage of 18.2 M reads per sample (range 13.5M-28.6 M). Per-gene counts were generated against the human reference transcriptome (Ensembl GRCh38 cDNA reference) using salmon (0.14.1). One sample pair failed quality control (Supplement C) and was removed. HGSOC transcriptomic subtypes were assigned using the consensusOv R package [23]. Significantly differentially expressed transcripts were identified using EdgeR [24]. Identified transcript clusters were mapped to significantly enriched pathways against the Molecular Signatures 2020 Database [21] using enrichR [22].

The Cancer Genome Atlas (TCGA) ovarian cancer data were accessed via the curatedOvarianData R package [25] (Supplement D).

### Quantification of immune cell infiltration

CD3 and CD8-positive cell infiltration were quantified by CD3 and CD8 IHC of whole 4 µm sections (Supplement E) (one slide of sarcomatous sample and one slide of carcinomatous sample for all 12 cases). Tumour areas were marked as regions of interest on digitised slides and positive tumour-infiltrating cells were quantified using the positive cell detection protocol in QuPath version 0.2.3. Automated counts were validated against two human observers in 22 images per marker (15% of samples), demonstrating excellent agreement between human and automated observers (spearman’s rho > 0.9 for all inter-observer comparisons, range 0.94–0.99).

Additional immune cell populations were investigated through deconvolution of RNA-sequencing data. The relative abundance of B cells, CD4 T cells, M1-like macrophages and M2-like macrophages were quantified using the consensusTME R package [26]; normalised per-gene read counts were used as the input. To assess the reliability of deconvoluted data, CD8 T cells were also quantified by this method and compared against immunohistochemistry-based quantification. There was a strong correlation between deconvolution- and immunohistochemistry-based quantification of CD8-positive cells (*r* = 0.74, *P* < 0.0001).

### Additional statistical analysis

All analyses were performed using R version 4.2.2. Continuous data were compared using the Mann–Whitney-*U*-test. Categorical data were compared using Barnard’s Test. Paired Mann–Whitney-*U* and Spearman’s rank association tests were used when analysing matched sarcomatous and carcinomatous samples. Statistical tests were two-sided; *P* < 0.05 was considered statistically significant unless otherwise specified.

## Results

### Genomic profiling of OCS

**Fig. 1:**
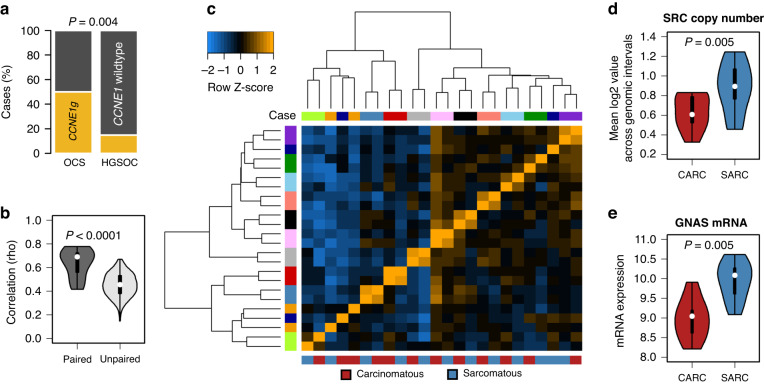
Genomic features of ovarian carcinosarcoma (OCS). **a** Comparison of *CCNE1* gain frequency between OCS and high grade serous ovarian carcinoma (HGSOC). **b** Quantified genomic copy number correlation between paired carcinomatous and sarcomatous samples, versus comparisons between all other samples. **c** Hierarchical clustering of copy number correlation matrix. Annotation bars are coloured by the patient (top) and sample type (bottom). **d** *SRC* copy number between carcinomatous and sarcomatous samples. **e** *GRAS* mRNA levels between carcinomatous and sarcomatous samples.

Eleven of the 12 sarcomatous samples contained shared *TP53* mutations within their respective carcinomatous sample pair (Table S2). The remaining pair did not contain a detectable *TP53* mutation. OCS cases demonstrated a high rate of *CCNE1* copy number gain (*CCNE1*g; 50.0%, 6 of 12 carcinomatous OCS samples), with a strong correlation in *CCNE1* copy number between the two compartments (Spearman’s rho 0.80, *P* < 0.0001) (Fig. S1). Comparison with a large cohort of HGSOC cases characterised by the same method [19] identified a significantly higher rate of *CCNE1*g in the carcinomatous compartments of OCS samples compared to HGSOC specimens (50.0%, 6 of 12 vs. 14.9%, 54 of 362 HGSOC, *P* = 0.004) (Fig. 1a).

**Fig. 2:**
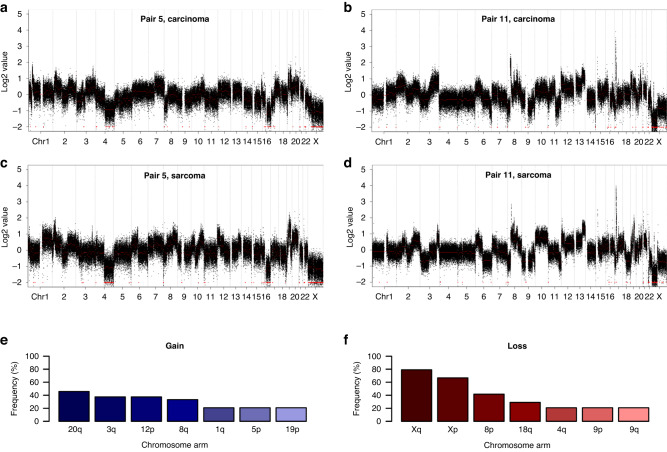
Global copy number landscape in ovarian carcinosarcoma (OCS). **a**, **b** Example global copy number profiles across 20kB genomic intervals in carcinomatous samples. The carcinomatous sample from pair 5 demonstrates evidence of loss on chromosome X, chromosome 4 and chromosome 16, among other events, while the carcinomatous sample from pair 11 shows focal gain on chromosome 17, among other events. **c**, **d** Global copy number profiles in matched sarcomatous samples from the same patients. **e** Recurrent chromosome arm-level gain events across OCS samples. **f** Recurrent chromosome arm-level loss events across all samples.

Loss of chromosome X was common across samples (67% with concurrent ChrXp and Xq loss), as was the loss of chr8p (42% of samples) (Fig. 2 and Fig. S2). The most common chromosome arm-level gain events occurred at chr3q, chr8q, chr12p and chr20q (38%, 33%, 38% and 46% of samples) (Fig. 2).

Correlation between global copy number estimates across 20 kB genomic intervals identified significantly stronger correlation between paired samples versus the wider sample population (median paired sample rho 0.69 vs 0.46 across all comparisons of unpaired samples, *P* < 0.0001) (Fig. 1b), suggesting that the carcinomatous sample from a given patient was more genomically similar to its sarcomatous pair compared to the carcinomatous samples from other patients, and vice versa for sarcomatous samples. Clustering of these data grouped samples predominantly by patient, rather than by sample type (carcinomatous vs sarcomatous) (Fig. 1c).

Analysis of genomic intervals with significant copy number differences between matched sarcomatous and carcinomatous samples revealed multiple genomic regions consistently altered between compartments (Table S3). These included two regions on chr20 containing *SRC* and *GNAS* that demonstrated consistent copy number gain in the sarcomatous component. Focussed analysis of genomic internals mapping to *SRC* demonstrated copy number gain in sarcomatous components (*P* = 0.0048) (Fig. 1d). Loci mapping to *GNAS* suggested higher copy number in the sarcomatous component, but this was not statistically significant (*P* = 0.0640) (Fig. S3); however, RNA-sequencing revealed significantly increased *GNAS* mRNA expression in sarcomatous samples (*P* = 0.0048) (Fig. 1e), which appeared limited to those with *GNAS* copy number gain (Fig. S3).

Within the TCGA HGSOC cohort, *GNAS* expression was significantly correlated with expression of the EMT markers Vimentin and N-cadherin, and the cancer cell stemness marker PROM1 (Table S4). Similarly, *SRC* expression was significantly correlated with expression of the EMT markers SNAIL and TGFβ, and the cancer cell stemness marker ALDH1 (Table S4).

### Transcriptomic landscape of OCS

**Fig. 3:**
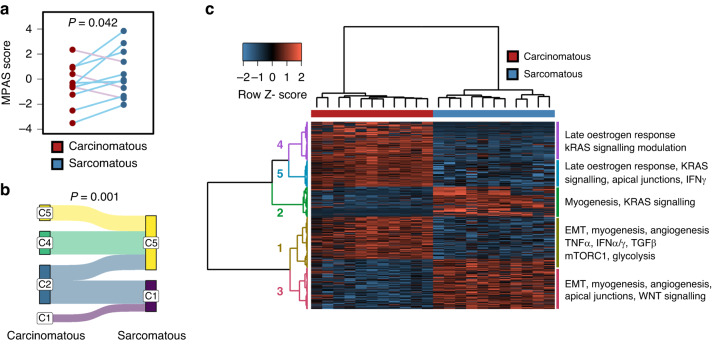
Transcriptomic features of ovarian carcinosarcoma (OCS). **a** MPAS transcriptomic score for MAPK activity between carcinomatous and sarcomatous samples. **b** Sankey plot showing assignment of carcinomatous and sarcomatous samples to transcriptomic subtypes derived in high grade serous ovarian carcinoma (HGSOC). **c** Hierarchical clustering of significantly differentially expressed genes between carcinomatous and sarcomatous samples identifies clusters of differentially expressed transcripts.

Sarcomatous samples demonstrated a significantly higher MPAS transcriptomic score for MAPK activity [27] compared to carcinomatous samples (*P* = 0.042) (Fig. 3a). Assignment of samples to transcriptomic subtypes derived from HGSOC showed the breadth of subtypes in the carcinomatous samples (*N* = 5 C2/IMR, *N* = 3 C4/DIF, *N* = 2 C5/PRO, *N* = 1 C1/MES). All cases with carcinomatous components of the favourable prognosis subtypes (C2/IMR and C4/DIF) transitioned toward poorer prognosis subtypes (C1/MES and C5/PRO) within the sarcomatous components (*N* = 4 C1/MES, *N* = 7 C5/PRO) (Fig. 3b). Accordingly, the poor prognosis subtypes (C1/MES, C5/PRO) were significantly enriched in sarcomatous compared to carcinomatous samples (*P* = 0.001).

While unsupervised transcriptomic analysis did not separate carcinomatous and sarcomatous samples with high fidelity (Fig. S4), we identified 1477 significantly differentially expressed transcripts between the carcinomatous and sarcomatous compartments. Hierarchical clustering demonstrated 5 clusters of differentially expressed genes (Fig. 3c). Clusters 4 and 5 represented genes more highly expressed in carcinomatous samples and were enriched for late oestrogen response genes. Cluster 3 comprised genes more highly expressed in sarcomatous samples and were significantly enriched for WNT signalling genes. Interferon signalling was enriched in clusters 5 and 1, both of which represented gene clusters more highly expressed in carcinomatous samples. Multiple clusters were enriched for EMT, myogenesis and apical junctions (Fig. 3c and Table S5). Multiple clusters were also mapped to modulation of KRAS signalling.

### MicroRNA expression profile in OCS

**Fig. 4:**
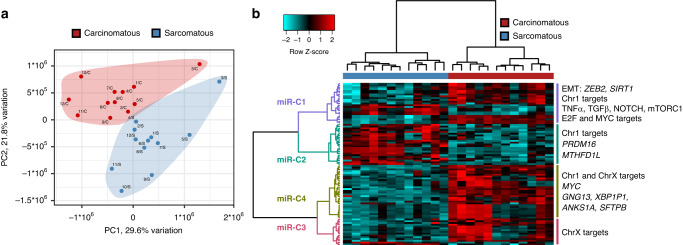
MicroRNA landscape in ovarian carcinosarcoma (OCS). **a** Principal component analysis demonstrates global differences in microRNA expression between compartments. **b** Hierarchical clustering of significantly differentially expressed microRNAs identifies clusters of differentially expressed microRNAs, targeting distinct genes and pathways.

Unsupervised analysis of microRNA expression demonstrated global differences in microRNA profiles between sarcomatous and carcinomatous samples, with samples separating by type upon principal component analysis (carcinomatous vs sarcomatous), rather than by patient (Fig. 4a). 91 significantly differentially expressed microRNAs were identified between the two compartments (absolute log fold-change >1.5, FDR < 0.01) (Table S6); clustering of these data identified four microRNA set clusters (Fig. 4b).

MicroRNA cluster 1 (miR-C1) demonstrated a large number of significantly enriched gene targets (*n* = 687), with the majority of microRNAs in this cluster being more highly expressed in carcinomatous samples. A number of genes were targeted by a large number of microRNAs in this cluster (>5 microRNAs), including the apoptosis and cell cycle-related genes *BLC2*, *CDK6* and *CDKN1B*, the cell signalling-associated genes *IGF1R*, *TGFBR1* and *PTEN*, and the EMT-related genes *SIRT1* and *ZEB2* (Table S7). Pathway analysis of miR-C1 target genes identified multiple significantly enriched pathways/processes (*P*-adj < 0.05), including EMT and apical junctions, alongside and E2F and MYC targets (Table S8). Gene targets were also significantly enriched for TNFα, TGFβ, mTORC1 and PI3K/AKT/mTOR signalling pathways (Fig. 4b).

MiR-C2 comprised microRNAs more highly expressed in sarcomatous samples, while miR-C3 and miR-C4 represented clusters of microRNAs more highly expressed in carcinomatous samples (Fig. 4b). MiR-C2 showed enrichment of the gene targets *PRFM16* and *MTHFD1L*, and were significantly enriched for chromosome X targets (*P*-adj = 0.0097). MiR-C3 showed no significantly enriched gene targets but demonstrated significant enrichment of chromosome X targets (*P*-adj < 0.0001), while miR-C4 demonstrated enrichment of five gene targets, including *MYC*, alongside targeting of chromosome X (*P*-adj < 0.0001) and chromosome 1 (*P*-adj = 0.004) (Fig. 4b and Table S9).

### Immune cell infiltration

**Fig. 5:**
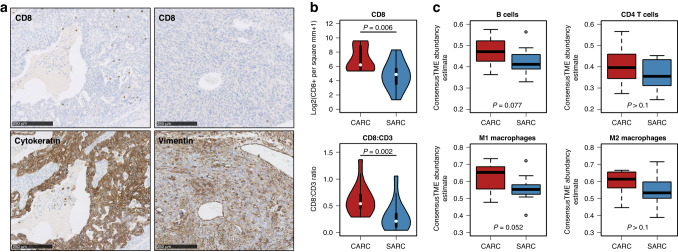
Tumour-infiltrating immune cell levels between carcinomatous and sarcomatous samples. **a** Example of images of CD8-positive cell infiltration (top panels) in vimentin-positive sarcomatous component (right panels) compared to matched cytokeratin-positive carcinomatous component (left panels). **b** Quantification of CD8-positive cells in carcinomatous and sarcomatous OCS samples and the ratio of CD8- and CD3-positive infiltrating cells between carcinomatous and sarcomatous OCS samples. **c** Relative abundance of B cells, CD4 T cells, M1-like macrophages and M2-like macrophages between carcinomatous and sarcomatous samples, as determined by deconvolution from bulk RNA-sequencing data.

The burden of infiltrating CD3+ and CD8+ cells, as assessed by immunohistochemistry, was highly heterogeneous across samples (Fig. S5). There was moderate-to-strong correlation between CD8-positive cell burden in the carcinomatous and sarcomatous compartments (spearman’s rho 0.67, *P* = 0.020) (Fig. S6); however, the sarcomatous samples contained significantly lower levels of CD8+ immune infiltration (*P* = 0.006) (Fig. 5a, b), with a lower ratio of CD8+:CD3+ cells (*P* = 0.002) (Fig. 5b and Fig. S7). Immune cell deconvolution from bulk RNA-sequencing data suggested that the carcinomatous component may also demonstrate a greater abundance of M1-like macrophages and B cells, though these differences did not pass the threshold for statistical significance (*P* = 0.052 and *P* = 0.077, respectively) (Fig. 5c).

## Discussion

OCS is a highly aggressive ovarian cancer type that demonstrates relative chemoresistance [1, 6, 7]. Recurrence rate and mortality are high across all stages and new treatment strategies to improve survival are urgently needed [1]. However, the identification of candidate targeted interventions has been hindered by a lack of fundamental understanding of molecular driver events [4]. To date, analysis of OCS samples has been limited to genomic profiling in small numbers of samples, most commonly by targeted sequencing [8]. There is a paucity of transcriptomic characterisation of OCS and a distinct lack of reports investigating other molecules of interest, such as microRNAs, or in determining levels of tumour engagement by the immune system.

Though previously hypothesised to represent collisions of independently formed tumours, we now recognise that OCS is of epithelial origin, with the sarcomatous compartment having formed through complete EMT, most commonly from HGSOC [2]. While OCS are therefore related to high grade ovarian carcinomas (≥95% from HGSOC or high grade endometrioid ovarian carcinoma [2, 4]), they are distinct in their clinical behaviour: OCS is diagnosed at a significantly older age, is significantly more chemoresistant, and is associated with significantly poorer survival despite a significantly higher rate of early-stage diagnosis compared to HGSOC [1, 6].

We identify shared *TP53* mutations between paired carcinomatous and sarcomatous samples, further supporting the notion of a shared clonal origin. We show that *CCNE1* copy number gain is common in OCS (50% in the carcinomatous samples) and that this frequency is significantly higher in OCS than in HGSOC; *CCNE1* gain may therefore predispose HGSOC to evolve into OCS. This is an interesting notion as *CCNE1* gain is a marker of poor prognosis in HGSOC [14, 19], may be associated with earlier stage at diagnosis (stage IV cases are reportedly depleted in *CCNE1*-gained HGSOC [19]), and is associated with older age at HGSOC diagnoses [28**–**30]; *CCNE1*-gained HGSOC may therefore represent a more OCS-like phenotype. Conversely, OCS may be conceptualised as an extreme form of *CCNE1*-gained HGSOC, though it is important to note that not all OCS harbour this event, and that not all OCS have carcinomatous components of high grade serous type [1]; *CCNE1* gain is not known to occur frequently in endometrioid ovarian carcinoma [12, 31, 32].

At the copy number level, paired carcinomatous and sarcomatous samples demonstrate substantial similarity; however, a number of specific genomic regions appear consistently altered between compartments. This includes a region containing *SRC* which is consistently gained in the sarcomatous component. *SRC* is widely implicated in EMT across a range of solid tumour types [33], and increased SRC activity via copy number gain is a feasible mechanism by which carcinomatous cells may dissociate cell-cell junctions, modulate the extracellular matrix, and transition toward a more mesenchymal phenotype [33]. *SRC* therefore represents a candidate driver of EMT to form the sarcomatous component, though it must be noted that functional investigations are required to validate this hypothesis, and that *SRC* is one of many genes encoded in the relevant region of chromosome 20. Another region consistently gained in the sarcomatous compartment contains *GNAS*; *GNAS* mRNA is significantly more highly expressed in the sarcomatous compartment compared to carcinomatous samples, supporting the notion that *GNAS* may be the driver gene within this amplicon. GNAS overexpression is associated with increased migration and proliferation in breast cancer models, while GNAS knockdown is able to inhibit EMT and breast tumour growth in vivo [34]. Increased *GNAS* expression associated with copy number gain may therefore represent a potential EMT driver in OCS, though - as with *SRC* - this hypothesis requires further investigation in model systems. Analysis of transcriptomic data from the TCGA HGSOC dataset suggests that *SRC* and *GNAS* expression may be associated with increased expression of EMT and cancer cell stemness markers, supporting the notion that these molecules may be able to drive EMT in ovarian cancers.

The transcriptomic landscape of OCS has remained poorly characterised to date. While unsupervised analysis of mRNA expression does not separate carcinomatous and sarcomatous samples with high fidelity, these compartments display a large number of significantly differentially expressed transcripts. Clusters of significantly differentially expressed genes are enriched for several hallmark processes with clear reflections of OCS biology, including EMT, myogenesis, and the apical junction complex. These analyses identify KRAS signalling modulation as significantly enriched across multiple gene clusters, alongside highlighting the potential importance of the WNT and TGFβ pathways in OCS. An mRNA score of RAS/MAPK pathway activity, the MPAS score [27], suggested increased MAPK activity within the sarcomatous component.

Beyond genomic and transcriptomic features, the microRNA landscape appears markedly different between the two compartments of OCS. Unsupervised analysis separates paired compartments to group samples by type rather than patient, suggesting consistent shifts in the global microRNA profiles between carcinomatous and sarcomatous regions. A large number of microRNAs are significantly differentially expressed between the two compartments, with clusters of deregulated microRNAs mapping to specific target genes. miR-C1 comprises microRNAs with a large number of significantly enriched gene targets, including the known EMT regulators *ZEB2* and *SIRT1*. SIRT1 is known to suppress TGFβ-mediated EMT in breast cancer and kidney epithelial cells [35]. High expression of microRNAs acting as negative SIRT1 regulators may therefore facilitate EMT in OCS. The TGFβ and NOTCH pathway are also significantly enriched within the gene targets of miR-C1.

Conversely, the other microRNA clusters are enriched for relatively few target genes. Only *PRDM16* and *MTHFD1L* are significantly enriched targets in miR-C2; miR-133, which is present in miR-C2, is reported to directly target and downregulate *PRDM16* to regulate fat cell differentiation [36], and PRDM16 overexpression has been reported to inhibit EMT in lung cancer models [37]. The high expression of miR-133 as part of miR-C2 within the sarcomatous component may therefore lead to suppression of *PRDM16* and subsequently facilitate sarcomatous component formation through EMT.

miR-C3 was enriched solely for chromosome X targets, while miR-C4 was enriched for chromosome X and chromosome 1 targets. While multiple clusters of differentially expressed microRNAs were significantly enriched for chromosome X targets, OCS samples also demonstrated a high frequency of chromosome X copy number loss, which affects both p and q arms in around two-thirds of samples. These data suggest that chromosome X is a crucial target of molecular events in OCS, disrupted through multiple distinct mechanisms at the genomic and microRNA levels. Chromosome X harbours a large number of functionally important genes, including tumour suppressors [38] and genes involved in drug metabolism and response [39]. Dysregulation of chromosome X gene expression has been investigated previously in ovarian carcinoma; however, much of this work has centred on escape from X-chromosome inactivation [40, 41]. Deletion of all or part of chromosome X has been reported in HGSOC and endometrioid ovarian carcinoma [42], but the functional significance of this event and the key target genes of these defects are poorly characterised.

Little is known about interactions between OCS and the tumour microenvironment. We show that, while infiltrating immune cells are evident in both sarcomatous and carcinomatous compartments, the sarcomatous compartment has significantly fewer infiltrating CD8-positive cells. This suggests a relative paucity of cytotoxic T cells. Levels of infiltrating cytotoxic T cells have been associated with prognosis across multiple tumour types, including ovarian cancer [43, 44]. The sarcomatous component’s apparent ability to better evade the cytotoxic T-cell response may contribute toward the poorer prognosis seen in this patient group. Alongside depleted CD8-positive cells, the sarcomatous component demonstrated lower levels of B cells and M1-like macrophages, though these differences did not reach the threshold for statistical significance. Together, these data suggest widespread differences in the tumour microenvironment and host immune response to the two separate malignant components, with the sarcomatous component more adept at evading this response. Immune checkpoint inhibitors may be a useful class of agents for reactivating the immune system against the sarcomatous component and could represent candidate treatment options for improving OCS patient outcomes. Currently, efficacy data for such agents in this disease context is extremely limited; no OCS cases were included in the phase 3 JAVELIN Ovarian 100 trial of first-line avelumab [45], and only 6 OCS cases were enroled in JAVELIN Ovarian 200 trial of avelumab in platinum-resistant ovarian cancer [46]. Case reports of pembrolizumab use in OCS have been made available in the literature, demonstrating that response to such agents is possible in OCS, but these reports are sparse and represent only anecdotal evidence [47, 48].

## Conclusion

We provide the first comprehensive molecular picture of OCS in a series of paired carcinomatous and sarcomatous samples. Gain of *CCNE1* and loss of chromosome X are common features of OCS. We identify shared and compartment-specific molecular events in these biphasic tumours, with features specific to the sarcomatous compartment representing potential EMT-associated events. *SRC* gain, increased *GNAS* expression and dysregulated microRNA expression represent potential mechanisms driving sarcomatous compartment formation by EMT that are worthy of further exploration, including functional investigation in model systems.

### Supplementary information

Supplementary information

## Data Availability

We are happy to provide all relevant data upon reasonable request to the corresponding author, subject to compliance with the relevant ethical framework.
