# Supplementary material for: Compartment-specific multiomic profiling identifies SRC and GNAS as candidate drivers of epithelial-to-mesenchymal transition in ovarian carcinosarcoma
Source: Br J Cancer. 2023 Dec 14;130(2):327–35. doi: 10.1038/s41416-023-02508-3 (PMC10803731; doi:10.1038/s41416-023-02508-3)
Supplement: Supplementary file 1 — Supplementary information [file 41416_2023_2508_MOESM1_ESM.docx]

**Compartment-specific multiomic profiling identifies SRC and GNAS as candidate drivers of epithelial to mesenchymal transition in ovarian carcinosarcoma**

**Supplement.**

**Supplement A: whole exome sequencing (extended methods)**

Whole exome libraries were prepared using the Illumina TruSeq Exome Library Prep kit (FC-150-1002) according to the manufacturer’s protocol, modified for DNA derived from formalin-fixed paraffin-embedded (FFPE) material.

300ng of DNA was sheared to an average fragment size of 150bp using the Covaris E220 Evolution Focused Ultra-Sonicator (Covaris Inc) and end-repaired to remove overhangs before fragment length optimisation using sample purification beads. A single 'A' nucleotide was added to the 3' ends of the blunt fragments to prevent ligation to other fragments during subsequent adapter ligation; a corresponding single 'T' nucleotide on the 3' end of the adapter provided a complementary overhang for adapter-fragment ligation. Multiple indexing adapters were ligated to the ends of the double-stranded cDNA to prepare them for hybridisation onto a flow cell, before 12 cycles of PCR were used to selectively enrich those DNA fragments that had adapter molecules on both ends and amplify the amount of DNA in the library suitable for sequencing.

Libraries were quantified using Qubit and the size distribution of fragments was assessed using the Agilent Bioanalyser. DNA libraries containing unique indexes were combined in pools of 12, and then target regions of the DNA were bound with capture probes. Streptavidin magnetic beads were then used to capture probes hybridised to the targeted regions of interest and a series of washes removed nonspecific binding from the beads. This process was repeated to ensure high specificity of the captured regions. Captured enriched library was then purified before 8 PCR amplification cycles and a final purification step.

Sequencing was performed on the NextSeq 2000 across two P3 flow cells. Sequencing quality was high (94.5% >Q30) with a mean of 109 million reads per samples (Range 43-189 million).

Exome sequencing data were processed using the bcbio nextgen high throughput sequencing workflow (v 1.2.4) for tumour-only sequencing: reads were aligned to the human reference genome (GRCh38) using bwa (v0.7.17), duplicates were marked prior to base quality score recalibration with GATK v4.1.9. The median per-sample on-target coverage was 109X.

Variant calling was performed using a majority vote system from three variant callers (VarDict 2019.06.04, Mutect2 and Freebayes 1.1.0.46); filtering for FFPE and oxidation artifacts was applied using DKFZBiasFilter. Called variants were annotated using the ensembl variant effect predictor (VEP v102) and converted to MAF files using vcf2maf v1.6.19.

Annotated variants were filtered to identify functional variation: variants with <0.1 allele frequency or <8 supporting reads were filtered. Common variants (maximum AF in across background populations >0.05) were filtered, and known pathogenic and known benign variants were then annotated using ClinVar. For remaining unannotated variants, non-coding and synonymous variants were filtered, while frameshift, nonsense, splice site, translational start site and stop codon mutations were flagged as likely functional. Missense mutations predictor pathogenic by both polyphen and SIFT prediction tools were retained as likely functional; other missense changes were flagged as variants of unknown significance (VUS). In-frame variants of unknown pathogenicity were flagged as VUS.

Genome-wide CN data were derived from aligned BAM files using the CopywriteR R package to calculate relative CN estimates at 20kb genomic intervals [1]. Median relative log2 CN ratios of intervals spanning each chromosome arm were calculated; a median of 0.5 and -0.5 were used as thresholds for high-confidence chromosome arm-level gains and losses, respectively. Copy number estimates across genomic intervals were compared using spearman’s rank-sum test to produce a matrix of per-sample correlations; clustering analysis was performed using this correlation matrix. Recurrent copy number changes between sarcomatous and sarcomatous regions were identified using paired Mann-Whitney U tests of quantified copy number estimates at P<0.001. Standalone identified 20kb intervals without copy number alteration in nearby genomic segments (within 5MB) were flagged as likely false positives. Remaining identified regions with copy number differences were merged with neighbouring identified genomic intervals (within 5MB) to define copy number-altered regions of interest for investigating potential driver genes. Resulting genomic intervals of interest were annotated for genes using the biomaRt R package [2].

**Supplement B: microRNA profiling (extended methods)**

MicroRNA profiling was performed using the HTG Molecular EdgeSeq microRNA Whole Transcriptome Assay, profiling 2,083 human microRNA transcripts. Matched sarcomatous and carcinomatous samples were lysed directly into the HTG EdgeSeq target capture reaction during the library preparation workflow. Target capture was performed using HTG EdgeSeq chemistry, after which libraries were prepared by PCR reactions to add tags, barcodes and adapters for cluster generation and Illumina sequencing. Three human brain RNA control samples were included in the run to enable quality control. After HTG EdgeSeq AMPure cleanup, libraries were sequenced using the Illumina NextSeq 550, producing 93.7% Q30 sequencing data. The HTG EdgeSeq Parser was used to align FASTQ files to the probe list and produce raw per-target count data. The median total counts per sample was 13.6M (range 6.0-17.7M).

Unsupervised analysis of microRNA expression profiles was performed by principal component analysis. Significantly differentially expressed microRNAs between carcinomatous and sarcomatous compartments were identified using a paired design matrix within EdgeR with a false discovery rate of <0.01 and an absolute log fold-change >1.5. Hierarchical clustering was performed using Euclidean distance and Ward’s linkage to identify clusters of differentially expressed microRNAs. The optimal number of microRNA clusters was determined using the gap statistic [3]. microRNA clusters were annotated for significantly over-represented target genes (compared to the complete list of arrayed microRNAs) using miEAA version 2.0 [4] with a minimum hit requirement of 3 and a false discovery rate <0.05. Identified gene targets were mapped to significantly enriched pathways against the Molecular Signatures 2020 Database [5] using the enrichR R package [6] at an adjusted P-value of <0.05.

**Supplement C: transcriptomic profiling (extended methods)**

mRNA profiling was performed using RNA extracted from matched sarcomatous and carcinomatous samples. Extraction was performed using the Qiagen miRNeasy kit according to the manufacturer’s protocol. RNA yield was quantified using the Qubit broad range RNA assay. Library preparation was performed with 500ng total input RNA using the Lexogen Quantseq 3’ mRNA-Seq Library Preparation Kit FWD for Illumina. Library generation was initiated by oligo-dT priming with primers already containing Illumina-compatible linker sequences. After first strand synthesis, RNA was removed and second strand synthesis initiated by random priming, after which samples underwent purification using magnetic beads and amplified by 17 cycles of PCR that introduced sequences for cluster generation and indexing. A final bead purification step was performed to remove adapter-dimers and excess primers prior to sequencing on an Illumina NextSeq 550. The mean coverage was 18.2M reads per sample (range 13.5M-28.6M).

Transcriptome data processed by clipping 12 bases from the 3’ end of each sequence using trimgalore (0.6.3) and counts were generated against the human reference transcriptome (Ensembl GRCh38 cDNA reference) using salmon (0.14.1). One sample pair failed quality control (3/S and 3/C) and was removed prior to analysis (figure S8).

Samples were assigned to transcriptomic subtypes derived from high grade serous ovarian carcinoma (C1/MES, C2/IMR C4/DIF, C5/PRO) using the Helland method within the ConsensusOv R package [7].

Significantly differentially expressed transcripts between sarcomatous and carcinomatous samples were identified using a paired design matrix in EdgeR [8] with a false discovery rate <0.01 and an absolute log fold-change >1.5.

Hierarchical clustering was performed using Euclidean distance and Ward’s linkage. Clusters of differentially expressed transcripts were mapped to significantly enriched pathways against the Molecular Signatures 2020 Database [5] using enrichR [6]; pathways with an adjusted P-value <0.05 and an odds ratio ≥3 were considered significant.

**Supplement D: investigation of TCGA ovarian cancer transcriptomic data (extended methods)**

Relationships between the expression of *SRC*/GNAS and markers of epithelial to mesenchymal transition (*SNAI1*, encoding SNAIL; *SNAI2*, encoding SLUG; *VIM*, encoding vimentin; *CDH2*, encoding N-Cadherin; *TFGB1*, encoding TGFβ) and cancer stem cells (*ALDH1A1*, encoding aldhehyde dehydrogenase A1; *PROM1*, encoding PROM1, also known as CD133) [9] were investigated using the TCGA ovarian cancer dataset. Transcriptomic data were accessed through the curatedOvarianData R package [10]; samples annotated as serous histology and high grade were identified (n=480) and filtered to retain samples with high tumour cell percentage (>80%) to minimise noise from stromal cells, leaving a cohort of 313 high purity HGSOC for analysis.

**Supplement E: CD3 and CD8-positive cell quantification (extended methods)**

T cell infiltration burden was quantified by CD3 and CD8 immunohistochemistry of whole 4µm sections on the Leica BOND III Autostainer using IHC protocol F. CD3 and CD8 IHC were performed using Leica ready-to-use CD3 (clone LN10) and CD8 (clone 4B11) mouse monoclonal antibodies. Stained slides were digitized using the Hamamatsu Nanozoomer at 40X magnification. Positive cells were quantified using Qupath version 0.2.3. For each tumour compartment, six 750um x 750um tumour-containing areas (minimum 25% tumour) were randomly selected using a custom R script; the tumour compartment was marked (guided by correspondingly H&E, cytokeratin, and vimentin-stained sections) as a region of interest and positive cells were quantified using the positive cell detection protocol. Automated scoring was validated using positive cell counting by two human observers (RLH, AO) in a subset of marked images for each marker (n=22 randomly selected images per marker, 15% of all images). Comparison of human and machine positive cell detection demonstrated excellent agreement (P<0.0001 and spearman’s rho>0.9 for all comparisons for both markers; range 0.94-0.98 for CD8, range 0.97-0.99 for CD3).

**Supplementary tables**

Table S1. Clinicopathological characteristics of molecularly profiled OCS cases

|  | | **N** | **%** |
| --- | --- | --- | --- |
| Age at diagnosis | Median | 66 | range 54-82 |
| Diagnosis period | Pre-2010 | 7 | 58 |
|  | 2010 onwards | 5 | 42 |
| FIGO stage at diagnosis | I | 1 | 8 |
|  | II | 1 | 8 |
|  | III | 10 | 83 |
|  | IV | 0 | 0 |
| Carcinomatous type | High grade serous | 12 | 100 |
| Heterologous sarcomatous elements identified | None (homologous) | 5 | 42 |
|  | Chondrosarcoma | 4 | 33 |
|  | Rhabdomyosarcoma | 3 | 25 |

Table S2. Summary mutational data on carcinosarcoma samples profiled by whole exome sequencing.

| **Category** | **Gene name** | **N samples with mutation** | **%** |
| --- | --- | --- | --- |
| Frequently mutated across cohort | *TP53* | 22 (one sample pair both wild-type) | 91.7 |
| Homologous recombination deficiency-related | *BRCA2* | 2 samples (identical mutation in paired samples) with missense mutation predicted damaging by polyphen and SIFT | 8.3 |
|  | *BRCA1* | 0 | 0 |
|  | *PABL2*, *BARD1*, *BRIP1*, *MRE11*, *NBN*, *EMSY*, RAD family genes, | 1 *RAD51* splice site mutation | 4.2 |
|  | FANC family genes | 1 *FANCM* missense mutation predicted damaging | 4.2 |
| Other common perturbed genes in HGSOC | *RB1* | 0 | 0 |
|  | *NF1* | 0 | 0 |
|  | *CCNE1* | 0 | 0 |
| Commonly perturbed genes in other ovarian cancer histotypes | *PIK3CA* | 1 missense mutation documented as pathogenic in ClinVar | 8.3 |
|  | *ARID1A* | 0 | 0 |
|  | *KRAS, NRAS, BRAF* | 0 | 0 |
|  | *PTEN* | 0 | 0 |
|  | *CTNNB1* | 0 | 0 |
|  | *USP9X* | 0 | 0 |
|  | MMR genes | 0 | 0 |
|  | MAPK-associated genes | 2 *MAP2K1* missense mutations predicted damaging by polyphen and SIFT (identical mutation in paired samples)  2 *MAP3K8* missense mutations predicted damaging by polyphen and SIFT (identical mutation in paired samples) | 16.7 |

Table S3. Identified copy number-altered genomic intervals between carcinomatous and sarcomatous samples

| **Chromosome** | **Start position** | **End position** |
| --- | --- | --- |
| chr1 | 34680001 | 40920000 |
| chr1 | 46160001 | 52800000 |
| chr1 | 75680001 | 77920000 |
| chr1 | 111860001 | 112220000 |
| chr1 | 145840001 | 152000000 |
| chr1 | 162880001 | 172220000 |
| chr3 | 62240001 | 69280000 |
| chr3 | 75580001 | 77220000 |
| chr3 | 108640001 | 109080000 |
| chr9 | 37180001 | 38660000 |
| chr10 | 4060001 | 4600000 |
| chr10 | 10780001 | 11020000 |
| chr10 | 20380001 | 20700000 |
| chr10 | 103120001 | 105260000 |
| chr10 | 116080001 | 116260000 |
| chr10 | 126240001 | 132620000 |
| chr13 | 93560001 | 96040000 |
| chr15 | 94400001 | 95340000 |
| chr16 | 76140001 | 78040000 |
| chr17 | 28160001 | 36560000 |
| chr18 | 28540001 | 32540000 |
| chr19 | 31160001 | 37660000 |
| chr20 | 31320001 | 41960000 |
| chr20 | 55880001 | 60300000 |
| chrX | 440001 | 5240000 |
| chrX | 12580001 | 31120000 |
| chrX | 37300001 | 40400000 |
| chrX | 46420001 | 53420000 |
| chrX | 64400001 | 65200000 |
| chrX | 70880001 | 127100000 |
| chrX | 136280001 | 138580000 |
| chrX | 148380001 | 155960000 |

Table S4. Correlation of *SRC* and *GNAS* expression with markers of epithelial to mesenchymal transition and cancer cell stemness in TCGA high grade serous ovarian cancer transcriptomic data

|  | | | ***SRC*** | | ***GNAS*** | |
| --- | --- | --- | --- | --- | --- | --- |
|  | **Protein** | **Transcript** | **Correlation (R)** | **P-value** | **Correlation (R)** | **P** |
| **EMT markers** | Snail | *SNAI1* | 0.21 | <0.001 | -0.02 | 0.745 |
|  | Slug | *SNAI2* | 0.07 | 0.271 | -0.01 | 0.823 |
|  | Vimentin | *VIM* | 0.06 | 0.350 | 0.42 | <0.001 |
|  | N-cadherin | *CDH2* | 0.06 | 0.322 | 0.14 | 0.017 |
|  | TGF-β | *TGFB1* | 0.15 | 0.012 | 0.03 | 0.624 |
| **Cancer cell stemness markers** | ALDH1 | *ALDH1A1* | 0.17 | 0.005 | 0.11 | 0.072 |
|  | PROM1/ CD133 | *PROM1* | 0.13 | 0.025 | 0.18 | 0.002 |

Table S5. Significantly enriched pathways in gene expression clusters

| **Cluster** | **Term** | **P-adj** | **Odds ratio** |
| --- | --- | --- | --- |
| 1 | Epithelial Mesenchymal Transition | <0.00001 | 6.38 |
| 1 | Interferon Alpha Response | <0.00001 | 8.57 |
| 1 | Interferon Gamma Response | <0.00001 | 6.38 |
| 1 | TNF-alpha Signaling via NF-kB | <0.00001 | 5.38 |
| 1 | Hypoxia | 0.00033 | 3.81 |
| 1 | Mitotic Spindle | 0.00033 | 3.83 |
| 1 | Myogenesis | 0.00033 | 3.81 |
| 1 | UV Response Up | 0.00045 | 4.14 |
| 1 | Coagulation | 0.00229 | 3.92 |
| 1 | Angiogenesis | 0.00247 | 8.03 |
| 1 | Estrogen Response Early | 0.00247 | 3.21 |
| 1 | Glycolysis | 0.00247 | 3.21 |
| 1 | mTORC1 Signaling | 0.00247 | 3.21 |
| 1 | Apoptosis | 0.00484 | 3.32 |
| 1 | UV Response Dn | 0.00582 | 3.34 |
| 1 | Androgen Response | 0.02978 | 3.18 |
| 1 | Reactive Oxygen Species Pathway | 0.03102 | 4.41 |
| 1 | TGF-beta Signaling | 0.04116 | 3.97 |
| 2 | Myogenesis | <0.00001 | 15.56 |
| 2 | KRAS Signaling Dn | 0.00640 | 4.12 |
| 3 | Epithelial Mesenchymal Transition | <0.00001 | 7.93 |
| 3 | UV Response Dn | 0.00009 | 5.66 |
| 3 | Apical Junction | 0.00484 | 3.60 |
| 3 | Myogenesis | 0.00484 | 3.60 |
| 3 | Wnt-beta Catenin Signaling | 0.00486 | 8.27 |
| 3 | Angiogenesis | 0.01894 | 7.63 |
| 4 | Estrogen Response Late | 0.00001 | 5.91 |
| 4 | KRAS Signaling Up | 0.00992 | 3.76 |
| 4 | KRAS Signaling Dn | 0.02550 | 3.36 |
| 5 | Estrogen Response Early | <0.00001 | 8.97 |
| 5 | Estrogen Response Late | 0.00002 | 6.45 |
| 5 | Apical Junction | 0.00033 | 5.26 |
| 5 | KRAS Signaling Dn | 0.00033 | 5.26 |
| 5 | p53 Pathway | 0.00033 | 5.26 |
| 5 | Coagulation | 0.00058 | 6.11 |
| 5 | Interferon Gamma Response | 0.00597 | 4.13 |
| 5 | KRAS Signaling Up | 0.02208 | 3.57 |
| 5 | Apoptosis | 0.02687 | 3.80 |

Table S6. Differentially expressed microRNAs between carcinomatous and sarcomatous samples

| **microRNA ID** | **Log Fold-change** | **False discovery rate** |
| --- | --- | --- |
| miR-141-3p | -7.59 | <0.00001 |
| miR-200c-3p | -7.53 | <0.00001 |
| miR-141-5p | -5.30 | <0.00001 |
| miR-429 | -6.41 | <0.00001 |
| miR-200c-5p | -3.38 | <0.00001 |
| miR-200b-3p | -6.52 | <0.00001 |
| miR-200a-3p | -6.57 | <0.00001 |
| miR-199a-5p | 2.82 | <0.00001 |
| miR-200a-5p | -4.01 | <0.00001 |
| miR-200b-5p | -4.62 | <0.00001 |
| miR-203b-3p | -2.39 | <0.00001 |
| miR-222-5p | -2.40 | <0.00001 |
| miR-934 | -4.80 | <0.00001 |
| miR-622 | -2.53 | <0.00001 |
| miR-221-5p | -2.67 | <0.00001 |
| miR-214-5p | 2.76 | <0.00001 |
| miR-492 | -2.99 | <0.00001 |
| miR-199a-3p | 2.40 | <0.00001 |
| miR-205-5p | -5.51 | <0.00001 |
| miR-4802-5p | -2.03 | <0.00001 |
| miR-195-5p | -2.74 | <0.00001 |
| miR-205-3p | -2.27 | <0.00001 |
| miR-4789-3p | -2.64 | <0.00001 |
| miR-222-3p | -2.79 | <0.00001 |
| miR-135b-5p | -4.47 | <0.00001 |
| miR-503-5p | 2.73 | <0.00001 |
| miR-29c-3p | -2.53 | <0.00001 |
| miR-34a-3p | -1.52 | <0.00001 |
| miR-221-3p | -2.59 | <0.00001 |
| miR-504-5p | 1.86 | <0.00001 |
| miR-133a-3p | 5.76 | <0.00001 |
| miR-133b | 6.24 | <0.00001 |
| miR-34a-5p | -1.70 | <0.00001 |
| miR-135b-3p | -3.12 | <0.00001 |
| miR-208b-3p | 2.70 | <0.00001 |
| miR-1 | 4.79 | <0.00001 |
| miR-133a-5p | 3.29 | <0.00001 |
| miR-887-3p | 1.96 | <0.00001 |
| miR-514b-3p | -2.54 | <0.00001 |
| miR-503-3p | 2.17 | <0.00001 |
| miR-508-3p | -3.87 | <0.00001 |
| miR-508-5p | -3.13 | <0.00001 |
| miR-198 | 1.67 | <0.00001 |
| miR-199b-5p | 2.36 | <0.00001 |
| miR-29b-2-5p | -1.72 | <0.00001 |
| miR-29c-5p | -1.81 | <0.00001 |
| miR-206 | 8.06 | <0.00001 |
| miR-615-3p | 1.95 | 0.00001 |
| miR-513c-5p | -3.70 | 0.00001 |
| miR-506-3p | -3.23 | 0.00001 |
| miR-513a-5p | -3.84 | 0.00001 |
| miR-509-3-5p | -3.66 | 0.00001 |
| miR-513b-5p | -4.08 | 0.00001 |
| miR-509-5p | -3.26 | 0.00001 |
| miR-509-3p | -3.32 | 0.00002 |
| miR-514a-3p | -4.16 | 0.00003 |
| miR-21-5p | -1.79 | 0.00004 |
| let-7f-5p | -1.63 | 0.00004 |
| miR-29a-3p | -2.33 | 0.00004 |
| miR-29b-3p | -2.18 | 0.00005 |
| miR-514b-5p | -3.08 | 0.00006 |
| miR-449c-5p | -2.69 | 0.00007 |
| miR-449a | -3.54 | 0.00009 |
| miR-1253 | 2.25 | 0.00011 |
| let-7g-5p | -1.55 | 0.00011 |
| miR-128-3p | 1.92 | 0.00012 |
| miR-135a-5p | -2.77 | 0.00013 |
| miR-513b-3p | -1.93 | 0.00020 |
| miR-181a-3p | 2.19 | 0.00020 |
| miR-506-5p | -2.20 | 0.00023 |
| miR-181a-5p | 1.85 | 0.00025 |
| miR-138-5p | 2.54 | 0.00031 |
| miR-424-3p | 2.00 | 0.00031 |
| miR-449b-5p | -3.46 | 0.00033 |
| miR-202-3p | -2.83 | 0.00043 |
| miR-31-5p | -3.67 | 0.00047 |
| miR-514a-5p | -2.08 | 0.00055 |
| miR-129-2-3p | 2.52 | 0.00070 |
| miR-181b-5p | 1.82 | 0.00096 |
| miR-510-5p | -2.60 | 0.00115 |
| miR-188-5p | 2.29 | 0.00131 |
| let-7b-5p | -1.60 | 0.00251 |
| miR-507 | -2.67 | 0.00275 |
| miR-187-3p | -1.65 | 0.00368 |
| miR-362-5p | 1.94 | 0.00555 |
| miR-501-5p | 2.26 | 0.00556 |
| miR-196b-5p | 3.58 | 0.00670 |
| miR-202-5p | -2.81 | 0.00745 |
| miR-196a-5p | 2.75 | 0.00814 |
| miR-1305 | 1.54 | 0.00848 |
| miR-196b-3p | 1.65 | 0.00941 |

Table S7. Significantly enriched gene targets targeted by >5 microRNAs

| **Target** | **P-adj** | **# hits** | **microRNAs** |
| --- | --- | --- | --- |
| *BCL2* | <0.00001 | 11 | miR-181a-5p; miR-181b-5p; miR-195-5p; miR-200b-3p; miR-200c-3p; miR-205-5p; miR-21-5p; miR-29a-3p; miR-29b-3p; miR-29c-3p; miR-34a-5p |
| *E2F3* | <0.00001 | 11 | let-7b-5p; miR-128-3p; miR-141-3p; miR-195-5p; miR-199a-5p; miR-199b-5p; miR-200b-3p; miR-200c-3p; miR-21-5p; miR-221-3p; miR-34a-5p |
| *PTEN* | <0.00001 | 13 | miR-128-3p; miR-141-3p; miR-181a-5p; miR-181b-5p; miR-200a-3p; miR-200c-3p; miR-205-5p; miR-21-5p; miR-221-3p; miR-222-3p; miR-29a-3p; miR-29b-3p; miR-29c-3p |
| *SERPINH1* | <0.00001 | 9 | miR-199a-5p; miR-199b-5p; miR-200b-3p; miR-200c-3p; miR-221-3p; miR-222-3p; miR-29a-3p; miR-29b-3p; miR-29c-3p |
| *SIRT1* | <0.00001 | 8 | miR-128-3p; miR-181a-5p; miR-181b-5p; miR-199a-5p; miR-199b-5p; miR-200c-3p; miR-29c-3p; miR-34a-5p |
| *ZBTB5* | <0.00001 | 9 | let-7b-5p; let-7f-5p; let-7g-5p; miR-195-5p; miR-221-3p; miR-222-3p; miR-29a-3p; miR-29b-3p; miR-29c-3p |
| *ZEB2* | <0.00001 | 9 | miR-141-3p; miR-181a-5p; miR-200a-3p; miR-200b-3p; miR-200c-3p; miR-205-5p; miR-221-3p; miR-222-3p; miR-34a-5p |
| *TIAM1* | 0.00001 | 6 | let-7b-5p; miR-141-3p; miR-21-5p; miR-221-3p; miR-29c-3p; miR-31-5p |
| *FOS* | 0.00001 | 8 | miR-181a-5p; miR-181b-5p; miR-221-3p; miR-222-3p; miR-29a-3p; miR-29b-3p; miR-29c-3p; miR-34a-5p |
| *CCNT2* | 0.00001 | 9 | let-7b-5p; let-7f-5p; let-7g-5p; miR-195-5p; miR-200b-3p; miR-200c-3p; miR-29a-3p; miR-29b-3p; miR-29c-3p |
| *JAG1* | 0.00001 | 6 | let-7b-5p; miR-199a-5p; miR-199b-5p; miR-200c-3p; miR-21-5p; miR-34a-5p |
| *VEGFA* | 0.00002 | 10 | miR-195-5p; miR-199a-3p; miR-199a-5p; miR-200b-3p; miR-200c-3p; miR-205-5p; miR-21-5p; miR-29a-3p; miR-29b-3p; miR-29c-3p |
| *AKT2* | 0.00002 | 6 | let-7b-5p; let-7g-5p; miR-21-5p; miR-29a-3p; miR-29b-3p; miR-29c-3p |
| *DICER1* | 0.00003 | 8 | let-7b-5p; miR-195-5p; miR-200a-3p; miR-21-5p; miR-221-3p; miR-222-3p; miR-29a-3p; miR-29c-3p |
| *TUBB2A* | 0.00003 | 11 | let-7b-5p; let-7f-5p; let-7g-5p; miR-181a-5p; miR-181b-5p; miR-195-5p; miR-200b-3p; miR-200c-3p; miR-29a-3p; miR-29b-3p; miR-29c-3p |
| *CDK6* | 0.00004 | 11 | let-7b-5p; miR-195-5p; miR-200a-3p; miR-205-5p; miR-21-5p; miR-221-3p; miR-222-3p; miR-29a-3p; miR-29b-3p; miR-29c-3p; miR-34a-5p |
| *DYRK3* | 0.00006 | 8 | let-7b-5p; let-7f-5p; let-7g-5p; miR-128-3p; miR-195-5p; miR-221-3p; miR-222-3p; miR-34a-5p |
| *ETS1* | 0.00007 | 7 | miR-181a-5p; miR-181b-5p; miR-199a-5p; miR-200b-3p; miR-200c-3p; miR-221-3p; miR-222-3p |
| *RAB40C* | 0.00009 | 6 | let-7b-5p; let-7f-5p; let-7g-5p; miR-29a-3p; miR-29b-3p; miR-29c-3p |
| *TMTC3* | 0.00009 | 7 | let-7b-5p; let-7f-5p; let-7g-5p; miR-128-3p; miR-29a-3p; miR-29b-3p; miR-29c-3p |
| *QKI* | 0.00012 | 8 | let-7b-5p; miR-141-3p; miR-199a-3p; miR-200a-3p; miR-200b-3p; miR-200c-3p; miR-222-3p; miR-29a-3p |
| *CDV3* | 0.00013 | 7 | let-7b-5p; let-7f-5p; let-7g-5p; miR-141-3p; miR-195-5p; miR-200a-3p; miR-29c-3p |
| *TGFBR3* | 0.00013 | 7 | let-7b-5p; let-7f-5p; let-7g-5p; miR-128-3p; miR-181a-5p; miR-195-5p; miR-21-5p |
| *VCL* | 0.00013 | 6 | let-7b-5p; let-7f-5p; let-7g-5p; miR-195-5p; miR-222-3p; miR-34a-5p |
| *Chromo-some 1* | 0.00015 | 10 | miR-181a-5p; miR-181b-5p; miR-199a-3p; miR-199a-5p; miR-200a-3p; miR-200b-3p; miR-205-5p; miR-29b-3p; miR-29c-3p; miR-34a-5p |
| *SLC10A7* | 0.00017 | 7 | let-7b-5p; let-7f-5p; let-7g-5p; miR-181a-5p; miR-181b-5p; miR-221-3p; miR-222-3p |
| *HUWE1* | 0.00018 | 6 | let-7b-5p; miR-181a-5p; miR-181b-5p; miR-29a-3p; miR-29c-3p; miR-34a-5p |
| *SP1* | 0.00020 | 9 | let-7b-5p; let-7f-5p; miR-128-3p; miR-200b-3p; miR-200c-3p; miR-21-5p; miR-29b-3p; miR-29c-3p; miR-31-5p |
| *MCL1* | 0.00021 | 8 | miR-141-3p; miR-181a-5p; miR-181b-5p; miR-200a-3p; miR-29a-3p; miR-29b-3p; miR-29c-3p; miR-34a-5p |
| *RPL12* | 0.00021 | 6 | let-7b-5p; let-7g-5p; miR-141-3p; miR-200a-3p; miR-222-3p; miR-31-5p |
| *GATA6* | 0.00025 | 10 | let-7b-5p; miR-128-3p; miR-141-3p; miR-181a-5p; miR-181b-5p; miR-199a-5p; miR-199b-5p; miR-200a-3p; miR-200b-3p; miR-200c-3p |
| *TGFBR1* | 0.00026 | 6 | let-7b-5p; let-7f-5p; let-7g-5p; miR-128-3p; miR-181a-5p; miR-199a-5p |
| *BCL2L11* | 0.00029 | 8 | miR-181a-5p; miR-181b-5p; miR-195-5p; miR-200b-3p; miR-200c-3p; miR-221-3p; miR-222-3p; miR-34a-5p |
| *PHACTR2* | 0.00032 | 6 | miR-181a-5p; miR-195-5p; miR-21-5p; miR-29a-3p; miR-29b-3p; miR-29c-3p |
| *AKT3* | 0.00039 | 6 | miR-195-5p; miR-221-3p; miR-222-3p; miR-29a-3p; miR-29b-3p; miR-29c-3p |
| *LYN* | 0.00048 | 6 | let-7b-5p; let-7f-5p; let-7g-5p; miR-205-5p; miR-221-3p; miR-222-3p |
| *MXD1* | 0.00048 | 6 | let-7b-5p; let-7f-5p; let-7g-5p; miR-29a-3p; miR-29b-3p; miR-29c-3p |
| *PLAG1* | 0.00048 | 7 | miR-128-3p; miR-181a-5p; miR-181b-5p; miR-195-5p; miR-29a-3p; miR-29b-3p; miR-29c-3p |
| *CPEB3* | 0.00053 | 6 | let-7b-5p; miR-195-5p; miR-205-5p; miR-21-5p; miR-29a-3p; miR-34a-5p |
| *MYC* | 0.00054 | 8 | let-7b-5p; let-7f-5p; let-7g-5p; miR-21-5p; miR-222-3p; miR-29a-3p; miR-29b-3p; miR-34a-5p |
| *AFF4* | 0.00084 | 6 | let-7b-5p; miR-128-3p; miR-181a-5p; miR-181b-5p; miR-195-5p; miR-205-5p |
| *PIK3R1* | 0.00120 | 6 | miR-128-3p; miR-195-5p; miR-21-5p; miR-221-3p; miR-29a-3p; miR-29b-3p |
| *DDIT4* | 0.00137 | 6 | miR-181a-5p; miR-181b-5p; miR-199a-3p; miR-200b-3p; miR-200c-3p; miR-221-3p |
| *ZBTB37* | 0.00148 | 8 | let-7b-5p; let-7f-5p; let-7g-5p; miR-128-3p; miR-199a-5p; miR-199b-5p; miR-221-3p; miR-222-3p |
| *FMNL3* | 0.00167 | 6 | let-7b-5p; let-7f-5p; let-7g-5p; miR-29a-3p; miR-29b-3p; miR-29c-3p |
| *PPP2R2A* | 0.00171 | 6 | let-7b-5p; let-7f-5p; let-7g-5p; miR-221-3p; miR-222-3p; miR-31-5p |
| *AGO1* | 0.00194 | 7 | let-7b-5p; let-7f-5p; let-7g-5p; miR-195-5p; miR-29a-3p; miR-31-5p; miR-34a-5p |
| *NR6A1* | 0.00194 | 6 | let-7b-5p; let-7f-5p; let-7g-5p; miR-181a-5p; miR-181b-5p; miR-195-5p |
| *RAP2C* | 0.00194 | 6 | let-7b-5p; miR-141-3p; miR-195-5p; miR-200a-3p; miR-200b-3p; miR-200c-3p |
| *SMAD2* | 0.00235 | 6 | let-7g-5p; miR-128-3p; miR-181a-5p; miR-200a-3p; miR-200b-3p; miR-205-5p |
| *HMGA2* | 0.00277 | 7 | let-7b-5p; let-7f-5p; let-7g-5p; miR-181a-5p; miR-181b-5p; miR-29b-3p; miR-34a-5p |
| *YAE1D1* | 0.00277 | 6 | let-7b-5p; let-7f-5p; let-7g-5p; miR-29a-3p; miR-29b-3p; miR-29c-3p |
| *CDKN1B* | 0.00346 | 7 | let-7b-5p; miR-181a-5p; miR-200b-3p; miR-200c-3p; miR-221-3p; miR-222-3p; miR-34a-5p |
| *ITGA3* | 0.00373 | 6 | let-7b-5p; let-7f-5p; let-7g-5p; miR-199a-3p; miR-199a-5p; miR-199b-5p |
| *PMAIP1* | 0.00412 | 7 | let-7b-5p; let-7f-5p; let-7g-5p; miR-181a-5p; miR-181b-5p; miR-200b-3p; miR-200c-3p |
| *MDM4* | 0.00429 | 7 | let-7b-5p; let-7f-5p; let-7g-5p; miR-141-3p; miR-200a-3p; miR-21-5p; miR-34a-5p |
| *TUBB* | 0.00495 | 6 | let-7b-5p; miR-181a-5p; miR-181b-5p; miR-195-5p; miR-200b-3p; miR-200c-3p |
| *HSPA1B* | 0.00548 | 6 | let-7b-5p; miR-181a-5p; miR-181b-5p; miR-195-5p; miR-221-3p; miR-34a-5p |
| *NCOA3* | 0.00595 | 6 | let-7b-5p; let-7f-5p; let-7g-5p; miR-181a-5p; miR-181b-5p; miR-29b-3p |
| *HMGB1* | 0.00630 | 7 | let-7b-5p; let-7g-5p; miR-181b-5p; miR-200a-3p; miR-205-5p; miR-21-5p; miR-34a-5p |
| *TNFRSF10B* | 0.00648 | 6 | let-7b-5p; let-7g-5p; miR-141-3p; miR-200a-3p; miR-21-5p; miR-34a-5p |
| *ZNF805* | 0.00666 | 6 | let-7b-5p; miR-141-3p; miR-200a-3p; miR-221-3p; miR-222-3p; miR-31-5p |
| *TNPO1* | 0.00735 | 6 | let-7b-5p; miR-128-3p; miR-181a-5p; miR-181b-5p; miR-21-5p; miR-34a-5p |
| *DDX6* | 0.00789 | 6 | miR-128-3p; miR-221-3p; miR-222-3p; miR-29a-3p; miR-29b-3p; miR-29c-3p |
| *DDX3X* | 0.00826 | 6 | miR-181a-5p; miR-181b-5p; miR-195-5p; miR-199a-5p; miR-199b-5p; miR-21-5p |
| *MAZ* | 0.00928 | 6 | miR-181a-5p; miR-222-3p; miR-29a-3p; miR-29b-3p; miR-29c-3p; miR-34a-5p |
| *SHOC2* | 0.00928 | 7 | miR-128-3p; miR-181a-5p; miR-181b-5p; miR-195-5p; miR-200b-3p; miR-200c-3p; miR-34a-5p |
| *PEG10* | 0.01104 | 6 | let-7b-5p; let-7f-5p; let-7g-5p; miR-128-3p; miR-221-3p; miR-34a-5p |
| *ZBTB34* | 0.01240 | 6 | miR-141-3p; miR-195-5p; miR-200a-3p; miR-29a-3p; miR-29b-3p; miR-29c-3p |
| *TNRC6B* | 0.01250 | 7 | let-7b-5p; miR-181a-5p; miR-181b-5p; miR-195-5p; miR-21-5p; miR-222-3p; miR-31-5p |
| *YOD1* | 0.01316 | 7 | let-7b-5p; let-7f-5p; let-7g-5p; miR-181a-5p; miR-21-5p; miR-221-3p; miR-34a-5p |
| *CELF1* | 0.01839 | 7 | let-7b-5p; let-7f-5p; let-7g-5p; miR-141-3p; miR-200a-3p; miR-200b-3p; miR-200c-3p |
| *HMGA1* | 0.02402 | 6 | let-7b-5p; let-7f-5p; let-7g-5p; miR-128-3p; miR-195-5p; miR-222-3p |
| *PPP1R15B* | 0.02445 | 6 | let-7b-5p; let-7f-5p; let-7g-5p; miR-141-3p; miR-200a-3p; miR-221-3p |
| *KMT2D* | 0.02601 | 6 | let-7b-5p; let-7f-5p; let-7g-5p; miR-195-5p; miR-222-3p; miR-34a-5p |
| *PLAGL2* | 0.03021 | 6 | let-7b-5p; let-7f-5p; let-7g-5p; miR-128-3p; miR-205-5p; miR-31-5p |
| *IGF1R* | 0.03161 | 7 | let-7b-5p; let-7f-5p; let-7g-5p; miR-141-3p; miR-181b-5p; miR-21-5p; miR-34a-5p |
| *NUFIP2* | 0.03688 | 8 | miR-128-3p; miR-195-5p; miR-200b-3p; miR-200c-3p; miR-205-5p; miR-21-5p; miR-221-3p; miR-34a-5p |
| *HDGF* | 0.04651 | 6 | miR-141-3p; miR-195-5p; miR-222-3p; miR-29a-3p; miR-29b-3p; miR-29c-3p |

Table S8. Pathway analysis of significantly enriched microRNA gene targets of microRNA cluster 1

| **Term** | **Overlap** | **P-adj** | **Odds ratio** |
| --- | --- | --- | --- |
| UV Response Dn | 26/144 | <0.00001 | 6.40 |
| G2-M Checkpoint | 30/200 | <0.00001 | 5.14 |
| Epithelial Mesenchymal Transition | 30/200 | <0.00001 | 5.14 |
| TNF-alpha Signaling via NF-kB | 25/200 | <0.00001 | 4.13 |
| Apoptosis | 22/161 | <0.00001 | 4.56 |
| Apical Junction | 22/200 | <0.00001 | 3.56 |
| TGF-beta Signaling | 11/54 | 0.00001 | 7.29 |
| E2F Targets | 21/200 | 0.00003 | 3.37 |
| p53 Pathway | 21/200 | 0.00003 | 3.37 |
| Hypoxia | 20/200 | 0.00010 | 3.19 |
| PI3K/AKT/mTOR Signaling | 13/105 | 0.00029 | 4.03 |
| IL-2/STAT5 Signaling | 18/199 | 0.00075 | 2.84 |
| Estrogen Response Early | 18/200 | 0.00075 | 2.83 |
| UV Response Up | 14/158 | 0.00402 | 2.77 |
| mTORC1 Signaling | 16/200 | 0.00451 | 2.48 |
| Myc Targets V1 | 16/200 | 0.00451 | 2.48 |
| Inflammatory Response | 16/200 | 0.00451 | 2.48 |
| Allograft Rejection | 15/200 | 0.01090 | 2.31 |
| Unfolded Protein Response | 10/113 | 0.01463 | 2.75 |
| Mitotic Spindle | 14/199 | 0.02239 | 2.15 |
| Myogenesis | 14/200 | 0.02239 | 2.14 |
| Apical Surface | 5/44 | 0.03851 | 3.62 |
| Adipogenesis | 13/200 | 0.04210 | 1.97 |
| Glycolysis | 13/200 | 0.04210 | 1.97 |
| KRAS Signaling Up | 13/200 | 0.04210 | 1.97 |
| Notch Signaling | 4/32 | 0.04451 | 4.03 |
| Peroxisome | 8/104 | 0.04970 | 2.36 |

Table S9. Significantly enriched gene targets of microRNA clusters 2, 3, and 4.

| **microRNA cluster** | **Target** | **P-adj** | **# hits** | **microRNAs** |
| --- | --- | --- | --- | --- |
| 2 | Chromosome X | 0.00967 | 7 | miR-188-5p; miR-362-5p; miR-424-3p; miR-501-5p; miR-503-3p; miR-503-5p; miR-504-5p |
| 2 | *MTHFD1L* | 0.04559 | 4 | miR-133a-5p; miR-138-5p; miR-615-3p; miR-887-3p |
| 2 | *PRDM16* | 0.04559 | 3 | miR-133a-3p; miR-133b; miR-615-3p |
| 3 | Chromosome X | 0.00002 | 8 | miR-506-3p; miR-506-5p; miR-507; miR-508-5p; miR-510-5p; miR-513b-3p; miR-514b-3p; miR-934 |
| 4 | Chromosome X | <0.00001 | 12 | miR-221-5p; miR-222-5p; miR-508-3p; miR-509-3-5p; miR-509-3p; miR-509-5p; miR-513a-5p; miR-513b-5p; miR-513c-5p; miR-514a-3p; miR-514a-5p; miR-514b-5p |
| 4 | *GNG13* | 0.00335 | 3 | miR-513a-5p; miR-513b-5p; miR-513c-5p |
| 4 | *XBP1P1* | 0.00335 | 6 | miR-135b-5p; miR-200a-5p; miR-200b-5p; miR-449a; miR-449b-5p; miR-449c-5p |
| 4 | Chromosome 1 | 0.00377 | 9 | miR-135b-3p; miR-135b-5p; miR-200a-5p; miR-200b-5p; miR-205-3p; miR-29b-2-5p; miR-29c-5p; miR-34a-3p; miR-429 |
| 4 | *ANKS1A* | 0.03354 | 5 | miR-449a; miR-449b-5p; miR-509-3p; miR-513a-5p; miR-622 |
| 4 | *MYC* | 0.03354 | 8 | miR-135b-5p; miR-34a-3p; miR-429; miR-449a; miR-449c-5p; miR-513c-5p; miR-514b-5p; miR-622 |
| 4 | *SFTPB* | 0.03354 | 4 | miR-509-3-5p; miR-509-5p; miR-513c-5p; miR-514b-5p |

**Supplementary figures**


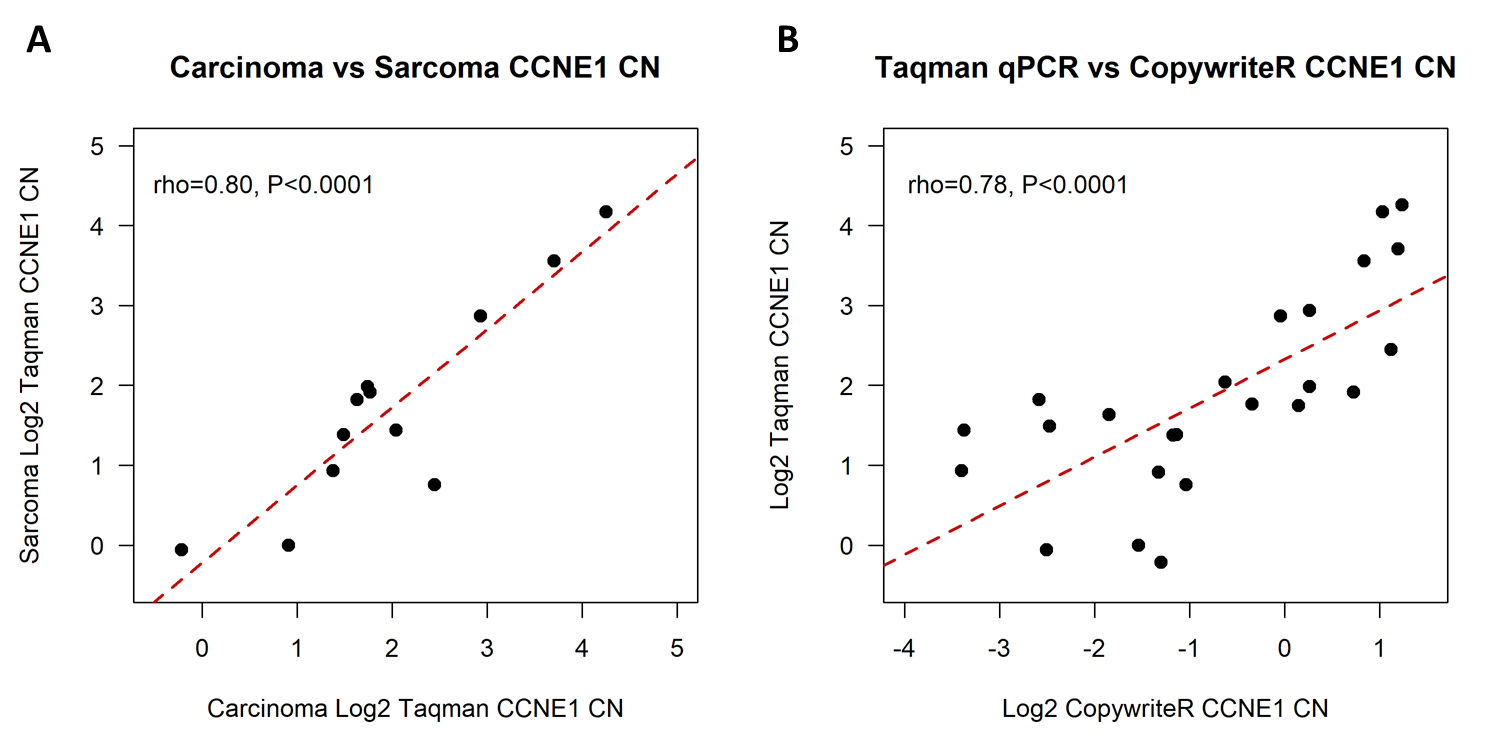


Figure S1. *CCNE1* copy number correlation. (A) *CCNE1* copy number determined by TaqMan qPCR between carcinomatous (n=12) and sarcomatous (n=12) samples. (B) *CCNE1* copy number determined by TaqMan qPCR compared to copywriteR genome-wide 20kB genomic interval copy number estimates


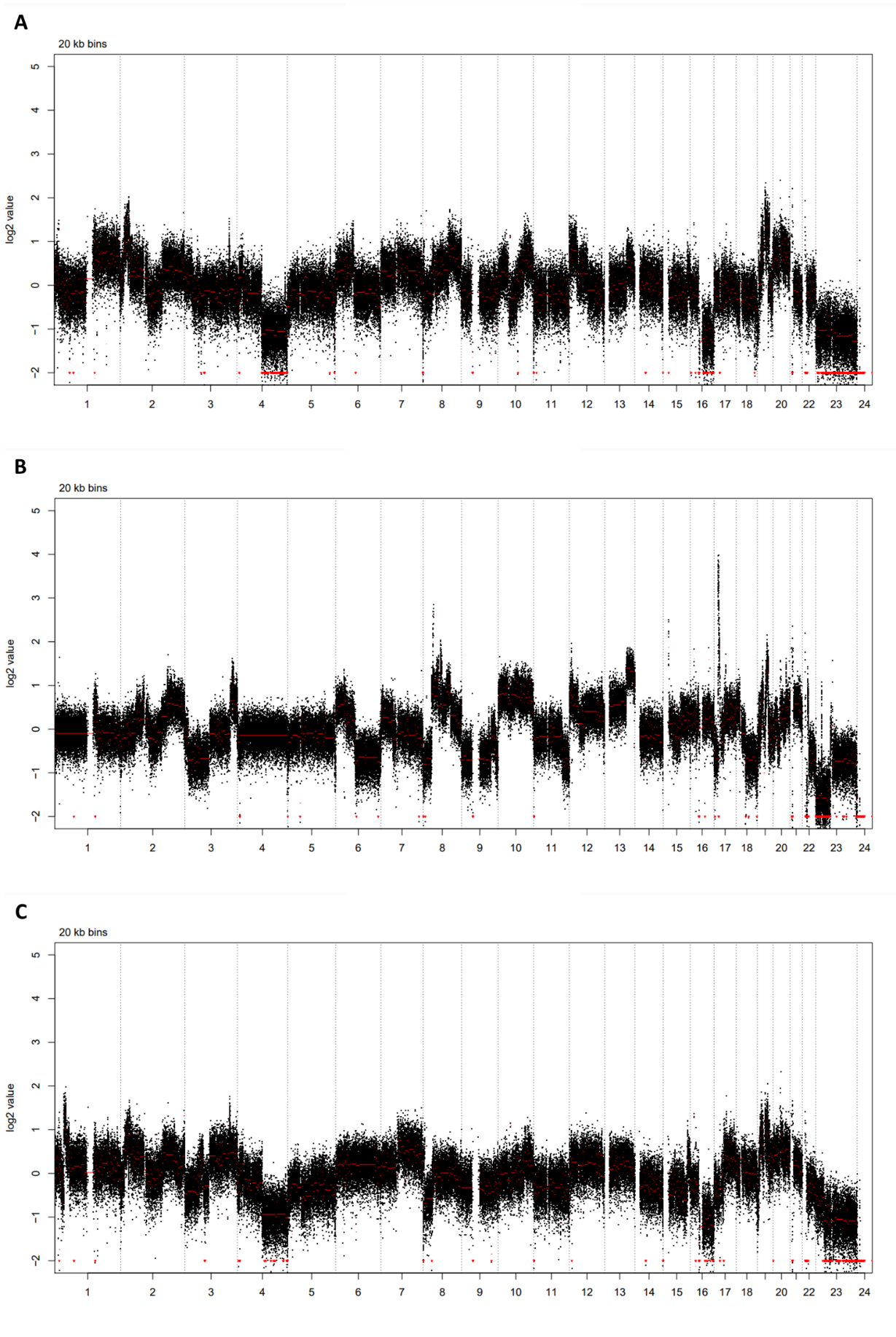


Figure S2. Visualized genome-wide copy number in OCS samples demonstrating recurrent chromosome-arm level copy number events. (A) Example case demonstrating loss of chromosome X (p and q), among other abnormalities (e.g. chr4q loss). (B) Example case with loss of chromosome X, among other abnormalities (e.g. chr10 gain). (C) Example case with loss of chromosome X and loss of 8p, among other abnormalities (e.g. 4q loss).


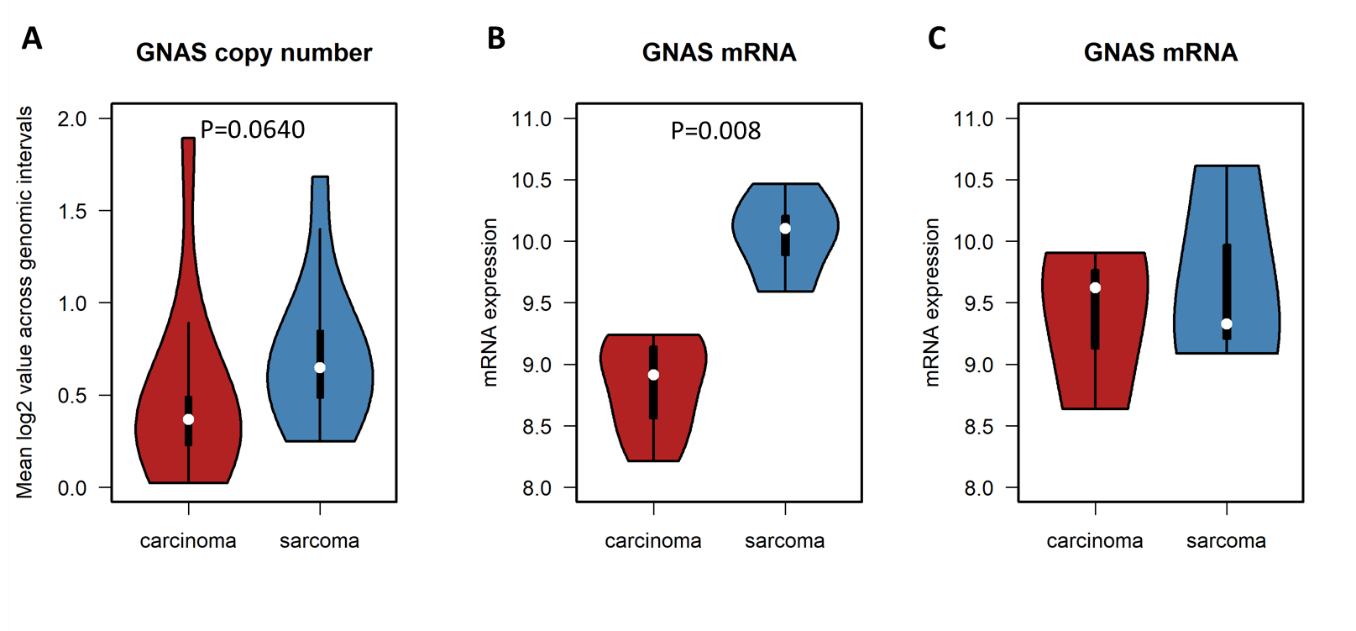


Figure S3. Comparison of *GNAS* copy number and mRNA expression between sarcomatous and carcinomatous samples. (A) *GNAS* copy number between compartments. (B) *GNAS* mRNA between compartments in cases where *GNAS* copy number increased in sarcomatous samples. (C) *GNAS* mRNA between compartments in cases where *GNAS* copy number did not increased in sarcomatous samples


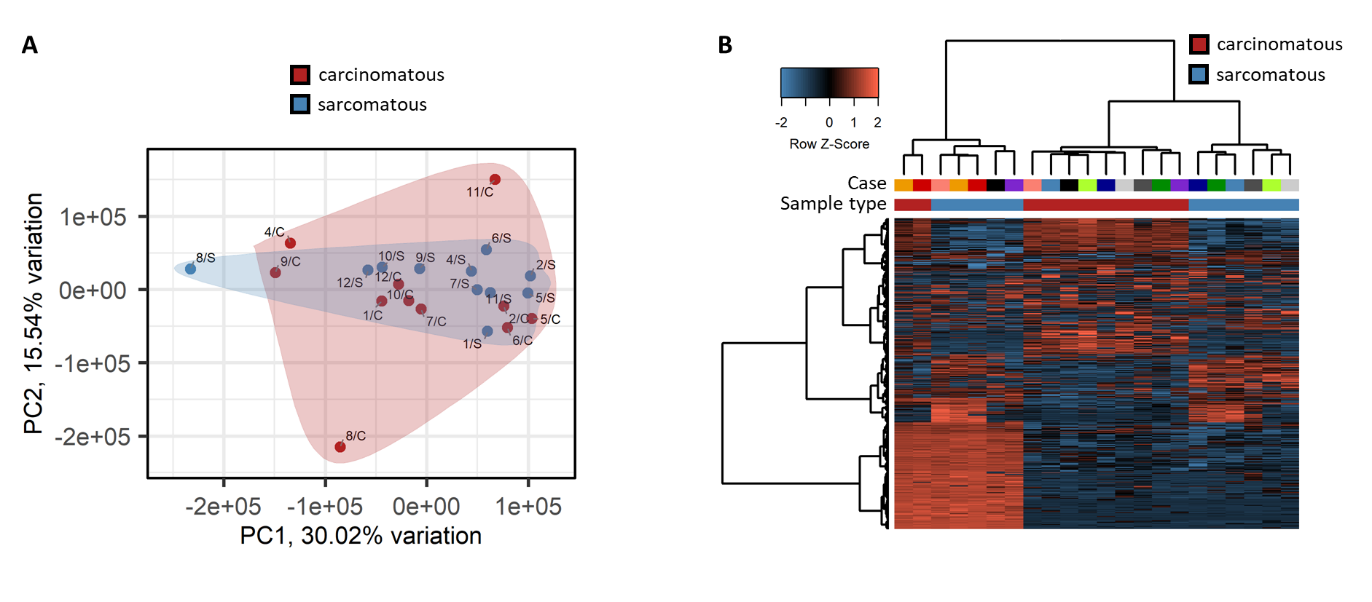


Figure S4. Unsupervised transcriptomic analysis. (A) Principal component analysis of mRNA expression data. (B) Unsupervised hierarchical clustering of mRNA expression data using top 1000 most variably expressed transcripts.


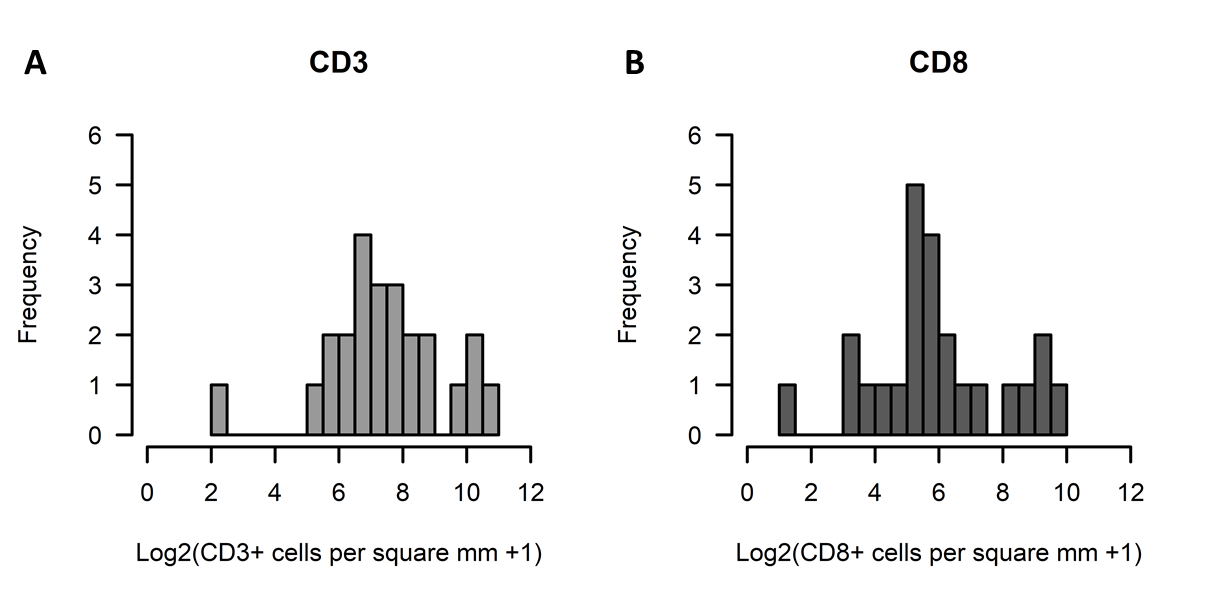


Figure S5. Histogram of CD3 and CD8 positive cell infiltration levels


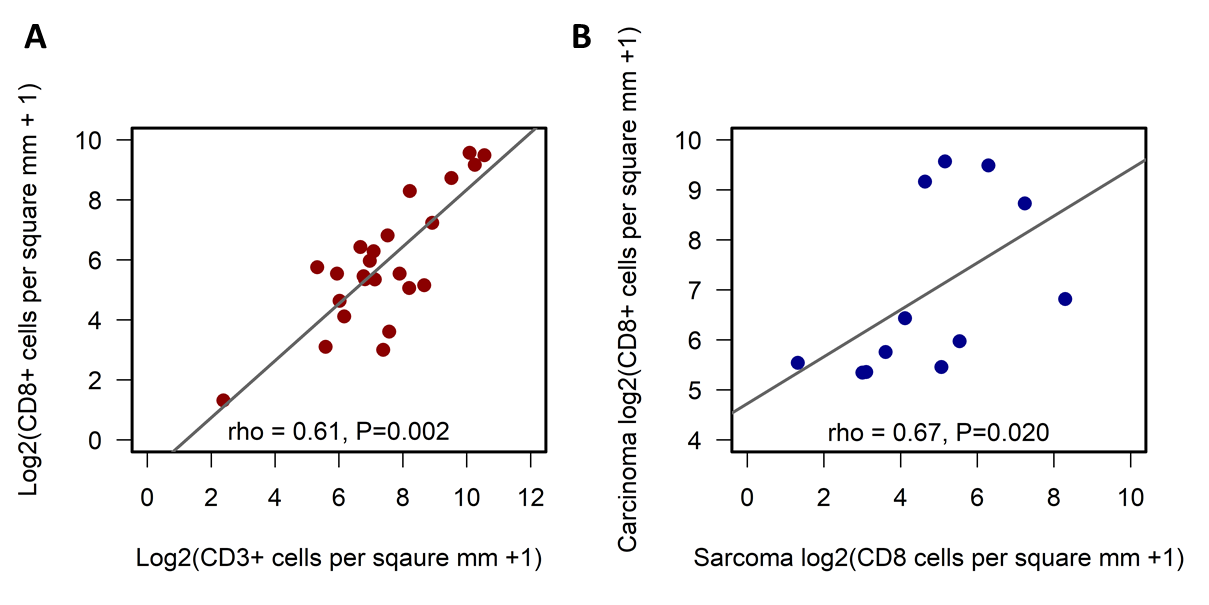


Figure S6. Correlation of CD3 and CD8-positive cell infiltration levels. (A) Comparison of CD3- and CD8-positive cell infiltration across all samples. (B) Comparison of CD8-positive cell infiltration between carcinomatous and sarcomatous samples


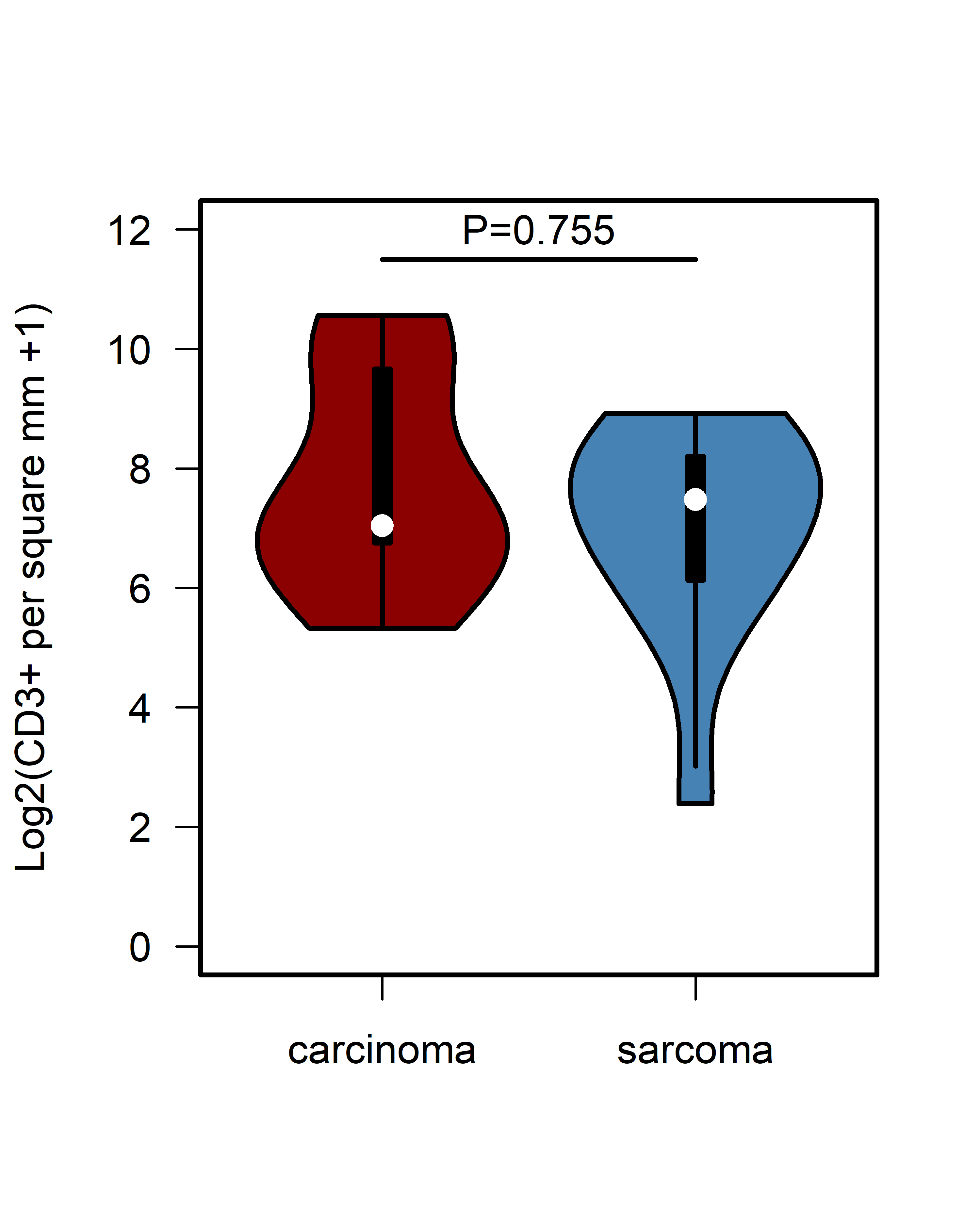


Figure S7. CD3-positive cell infiltration levels between carcinomatous and sarcomatous samples


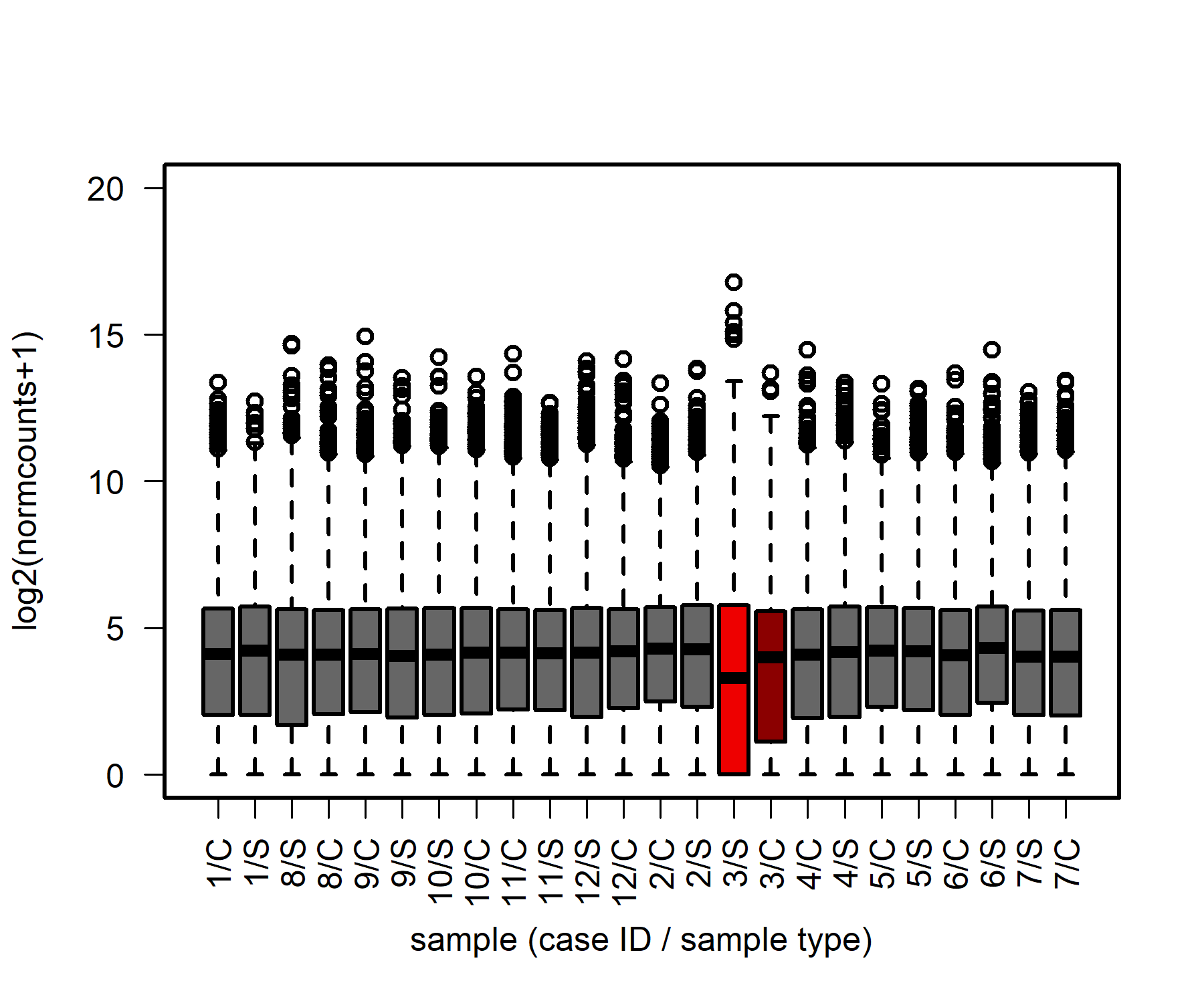


Figure S8. Quality control assessment of RNA-seq normalised read counts

**Supplementary references**

1. Kuilman, T., et al., *CopywriteR: DNA copy number detection from off-target sequence data.* Genome Biol, 2015. **16**(1): p. 49.

2. Durinck, S., et al., *BioMart and Bioconductor: a powerful link between biological databases and microarray data analysis.* Bioinformatics, 2005. **21**(16): p. 3439-40.

3. Kassambara, A. and F. Mundt, *factoextra: Extract and Visualize the Results of Multivariate Data Analyses*. 2020, <https://CRAN.R-project.org/package=factoextra>.

4. Kern, F., et al., *miEAA 2.0: integrating multi-species microRNA enrichment analysis and workflow management systems.* Nucleic Acids Res, 2020. **48**(W1): p. W521-w528.

5. Liberzon, A., et al., *Molecular signatures database (MSigDB) 3.0.* Bioinformatics, 2011. **27**(12): p. 1739-40.

6. Kuleshov, M.V., et al., *Enrichr: a comprehensive gene set enrichment analysis web server 2016 update.* Nucleic Acids Res, 2016. **44**(W1): p. W90-7.

7. Chen, G.M., et al., *Consensus on Molecular Subtypes of High-Grade Serous Ovarian Carcinoma.* Clin Cancer Res, 2018. **24**(20): p. 5037-5047.

8. Robinson, M.D., D.J. McCarthy, and G.K. Smyth, *edgeR: a Bioconductor package for differential expression analysis of digital gene expression data.* Bioinformatics, 2010. **26**(1): p. 139-40.

9. Burgos-Ojeda, D., B.R. Rueda, and R.J. Buckanovich, *Ovarian cancer stem cell markers: prognostic and therapeutic implications.* Cancer Lett, 2012. **322**(1): p. 1-7.

10. Ganzfried, B.F., et al., *curatedOvarianData: clinically annotated data for the ovarian cancer transcriptome.* Database (Oxford), 2013. **2013**: p. bat013.
